# Supplementary material for: Ionizing Radiation Induces Resistant Glioblastoma Stem-Like Cells by Promoting Autophagy via the Wnt/β-Catenin Pathway
Source: Life (Basel). 2021 May 18;11(5):451. doi: 10.3390/life11050451 (PMC8157563; doi:10.3390/life11050451)

Related to Fig.3D-1

Anti-P62

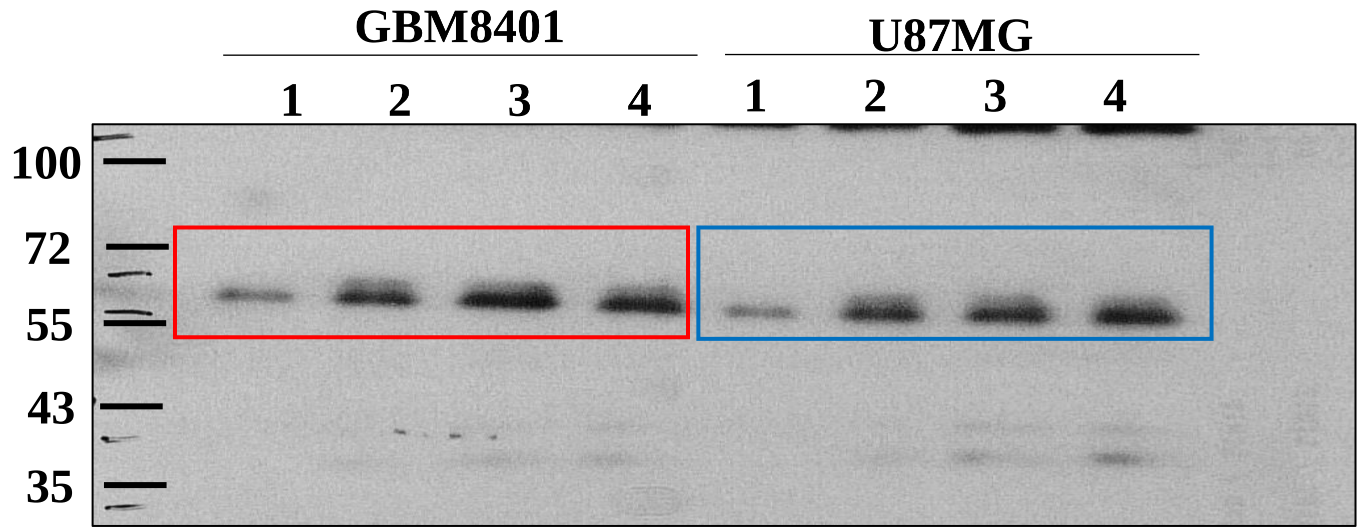

1 : 2.02 : 2.81 : 2.00   1 : 1.73 : 2.23 : 2.08

Anti-LC3 I/II

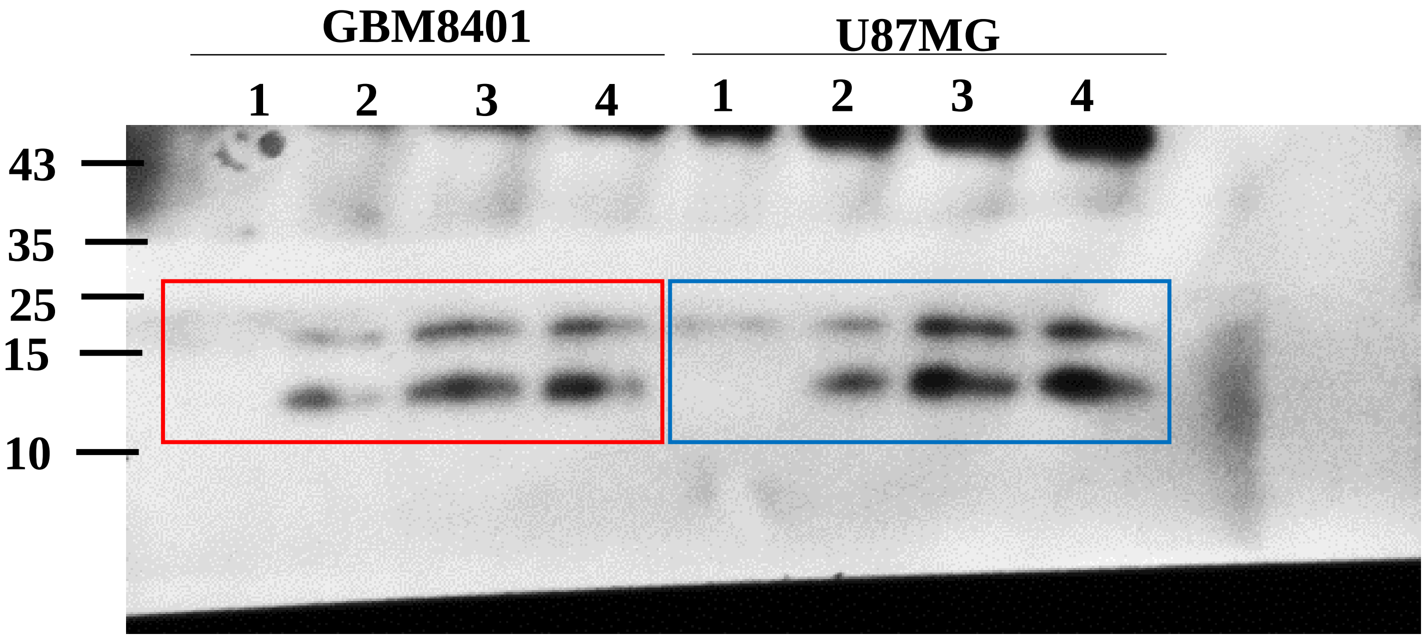

LC3II 1 : 2.94 : 5.23 : 4.74   1 : 2.71 : 4.09 : 4.13

Anti-Nestin

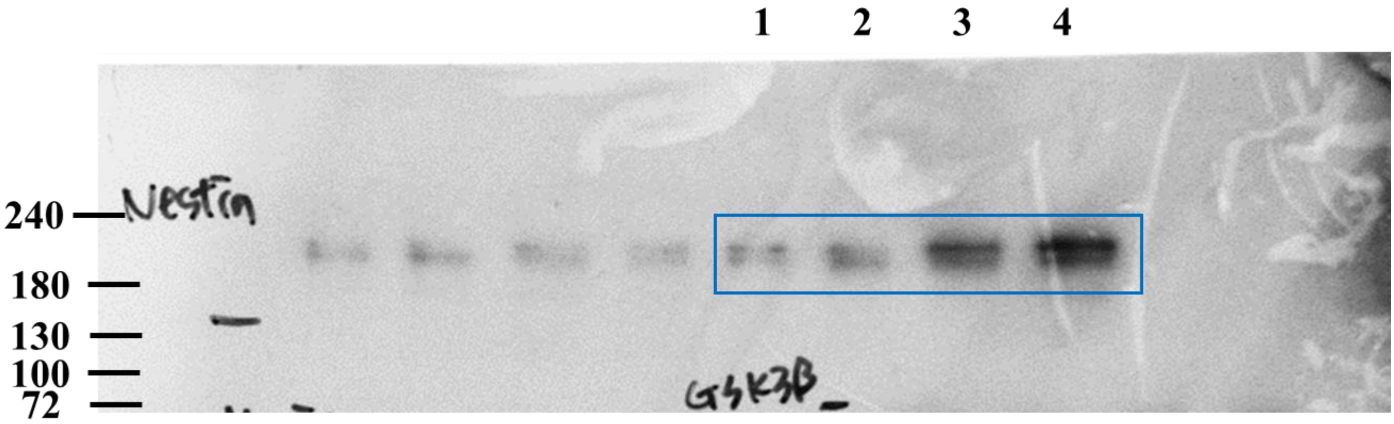

1 : 1.57 : 2.59 : 3.18

Related to Fig.3D-2

Anti-Nestin

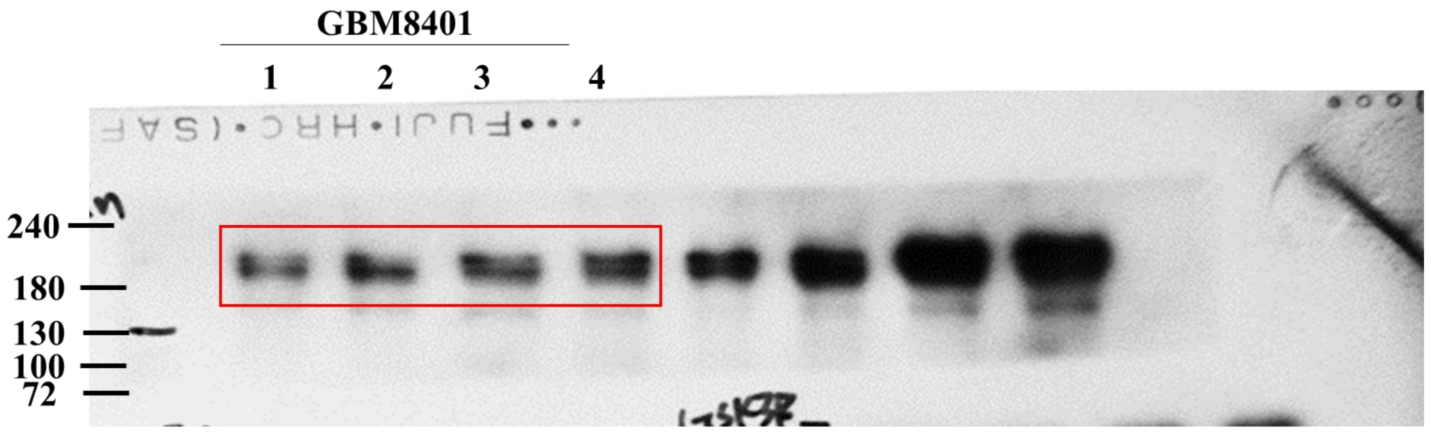

1 : 1.31 : 1.62 : 1.39

Anti-CD44

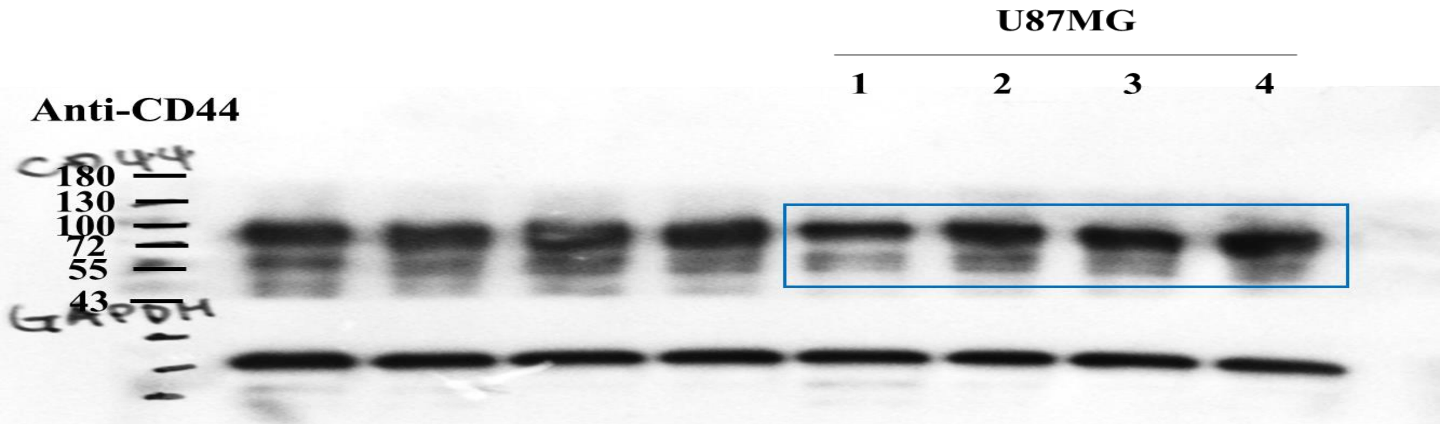

1 : 1.38 : 1.45 : 1.56

Anti-CD44

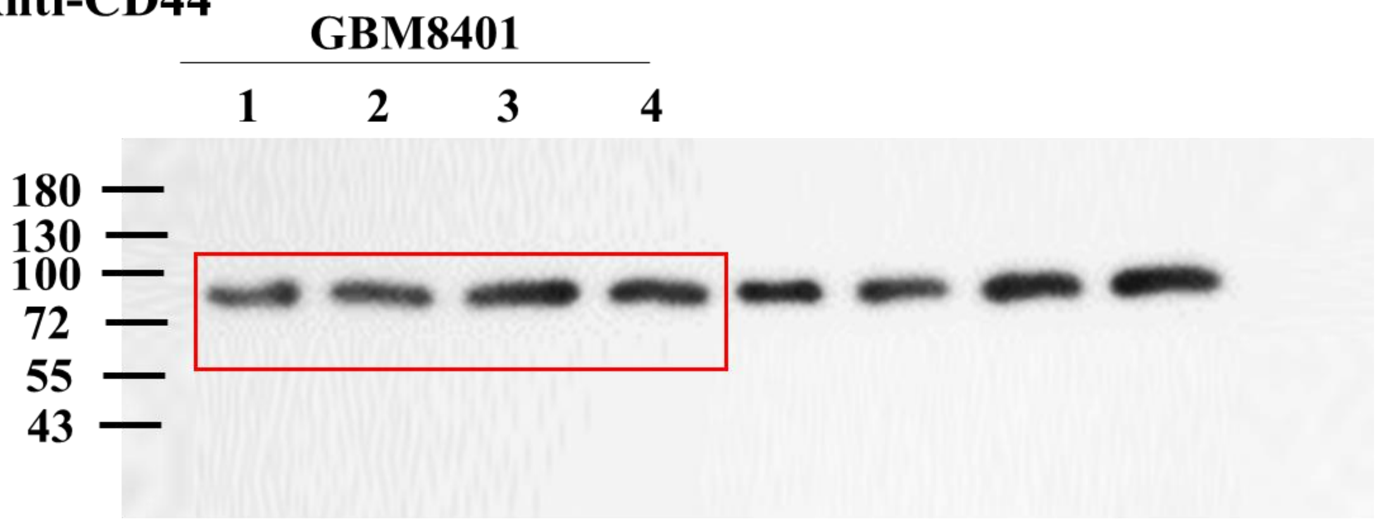

1 : 1.22 : 1.57 : 1.66

Related to Fig.3D-3

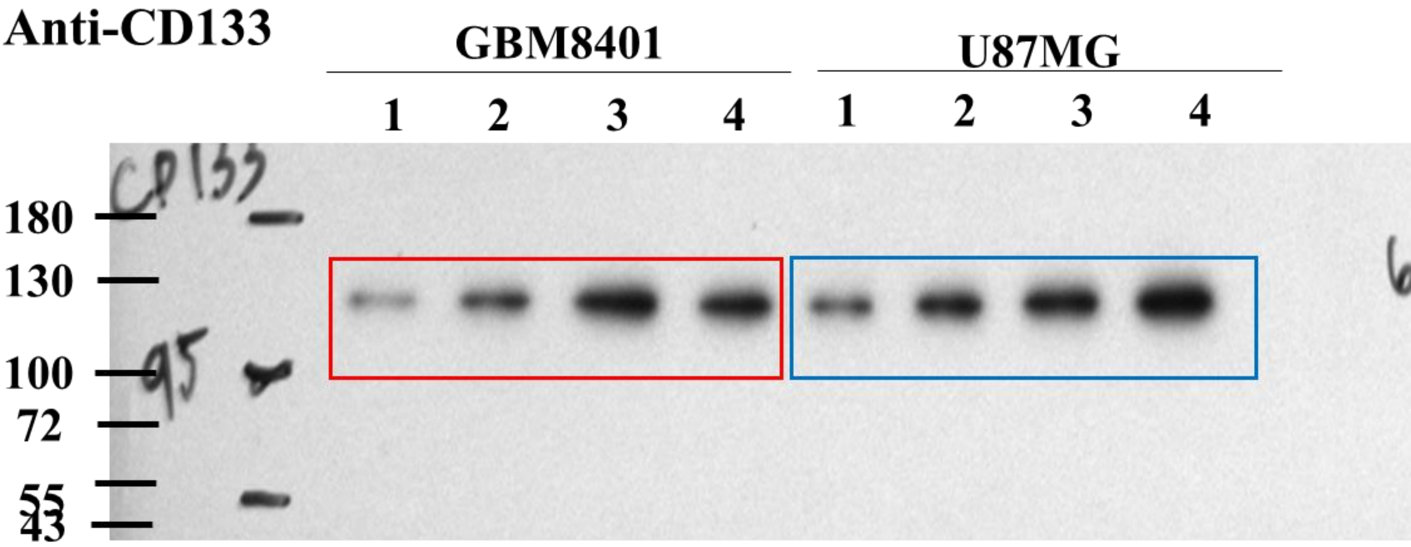

1 : 1.45 : 2.33 : 1.80   1 : 1.68 : 2.83 : 3.10

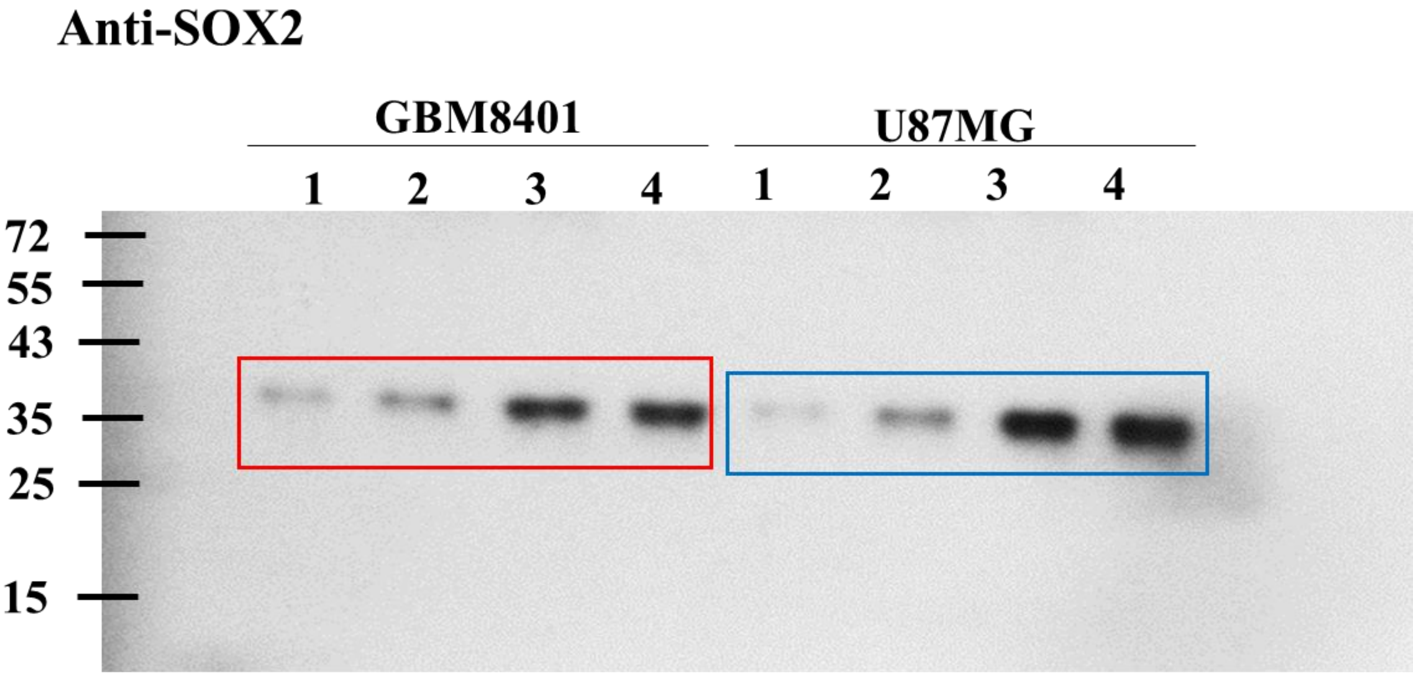

1 : 1.91 : 2.20 : 2.03   1 : 1.69 : 4.27 : 4.22

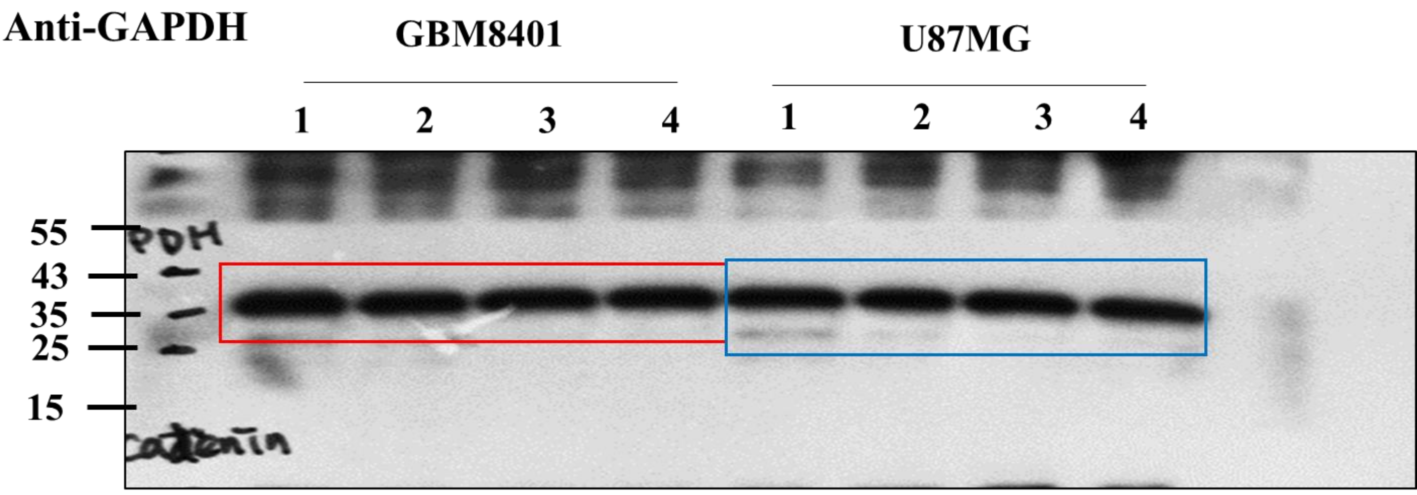

1 : 1.03 : 1.01 : 1.02   1 : 0.96 : 1.05 : 1.02

Related to Fig.4A-1

Anti-Frizzled

U87MG

1 2 3 4

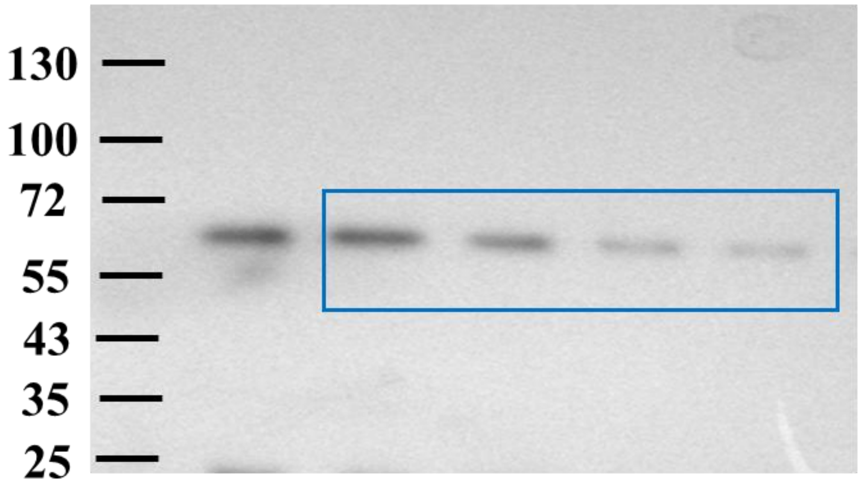

1 : 0.69 : 0.57 : 0.57

Anti-Frizzled

GBM8401

1 2 3 4

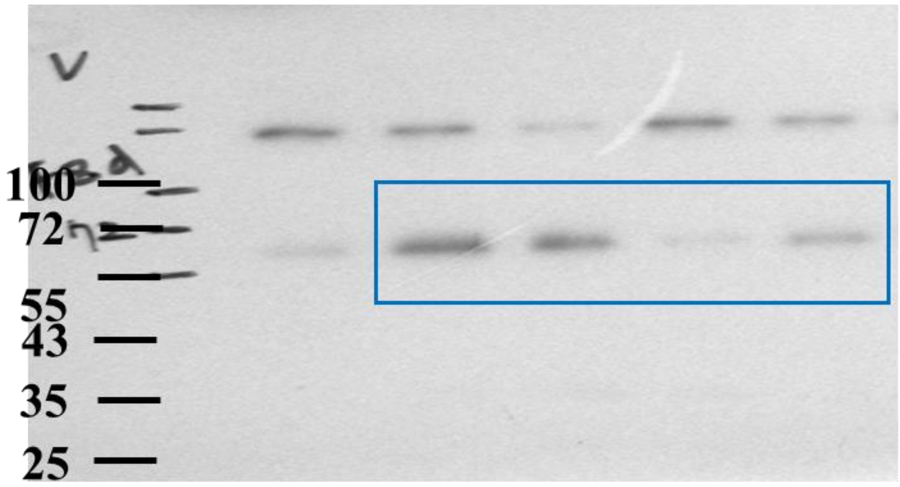

1 : 0.69 : 0.57 : 0.57

Anti-GSK3β

U87MG

1 2 3 4

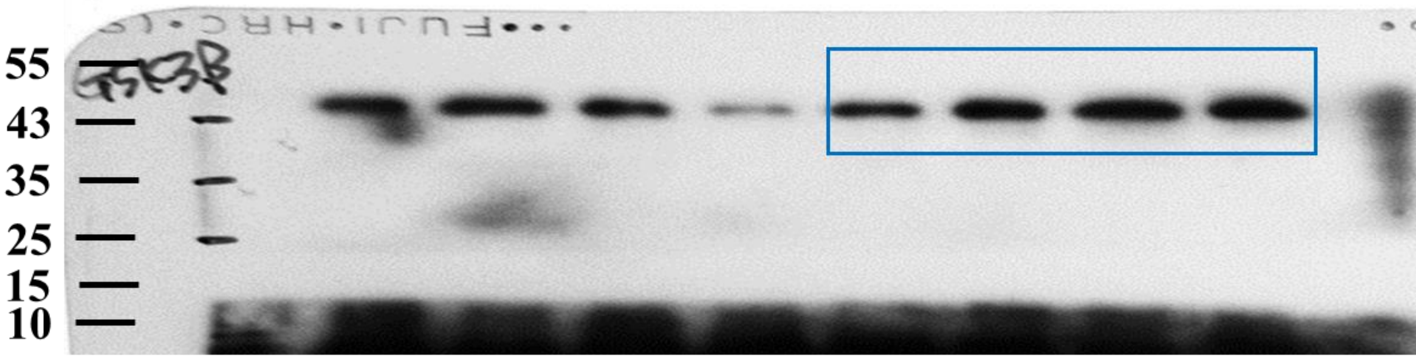

1 : 1.26 : 1.49 : 1.45

Related to Fig.4A-2

Anti-GSK3β

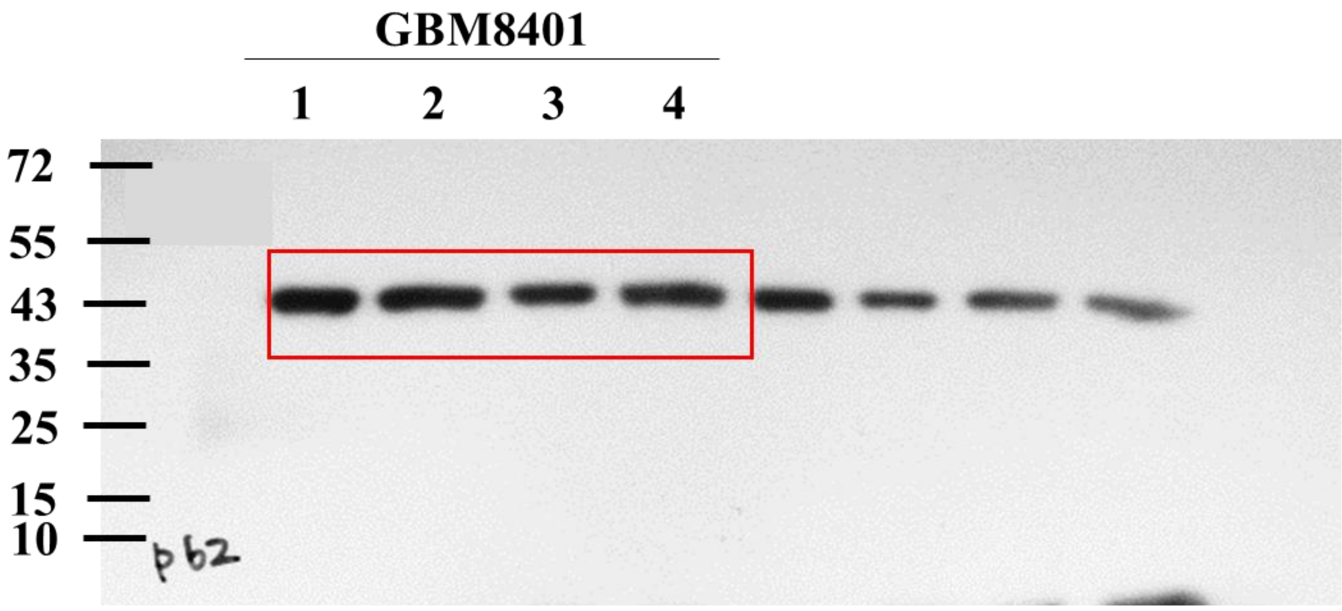

1 : 1.03 : 0.89 : 0.94

Anti-GSK3β (S9)

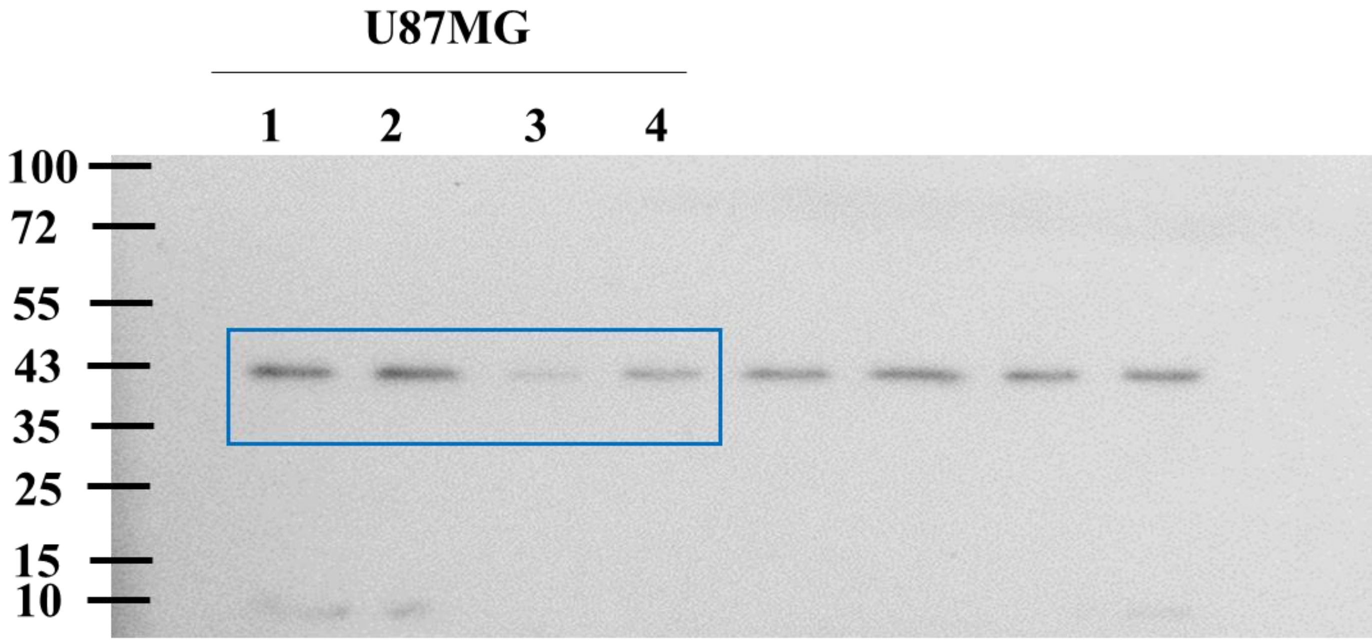

1 : 0.96 : 0.62 : 0.73

Anti-GSK3β (S9)

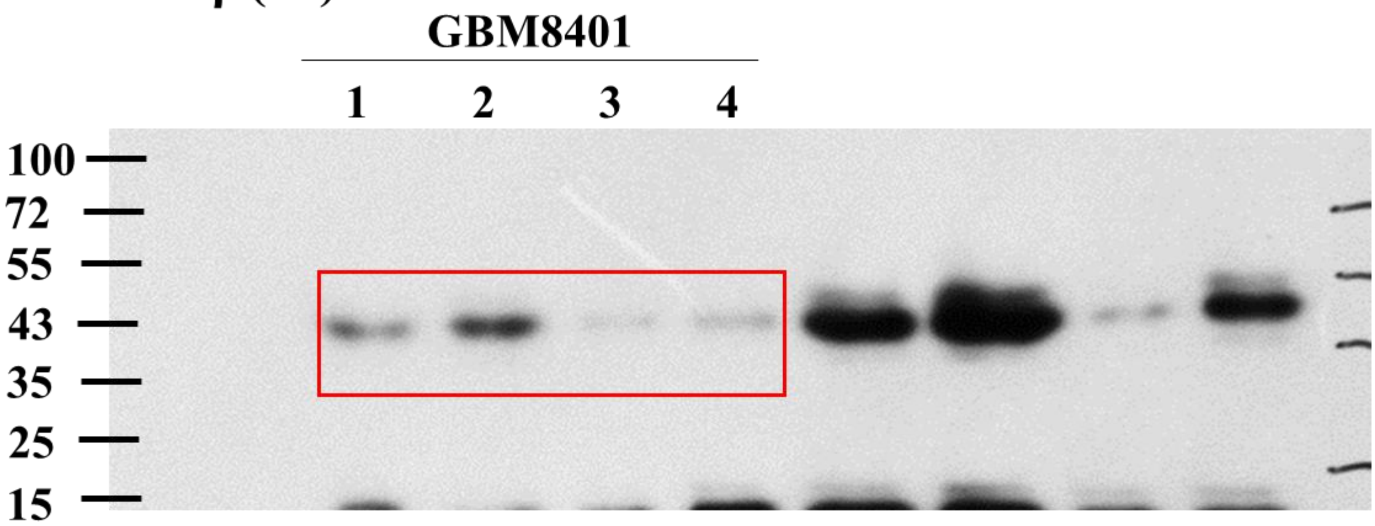

1 : 1.25 : 0.53 : 0.61

Related to Fig.4A-3

Anti-β-catenin

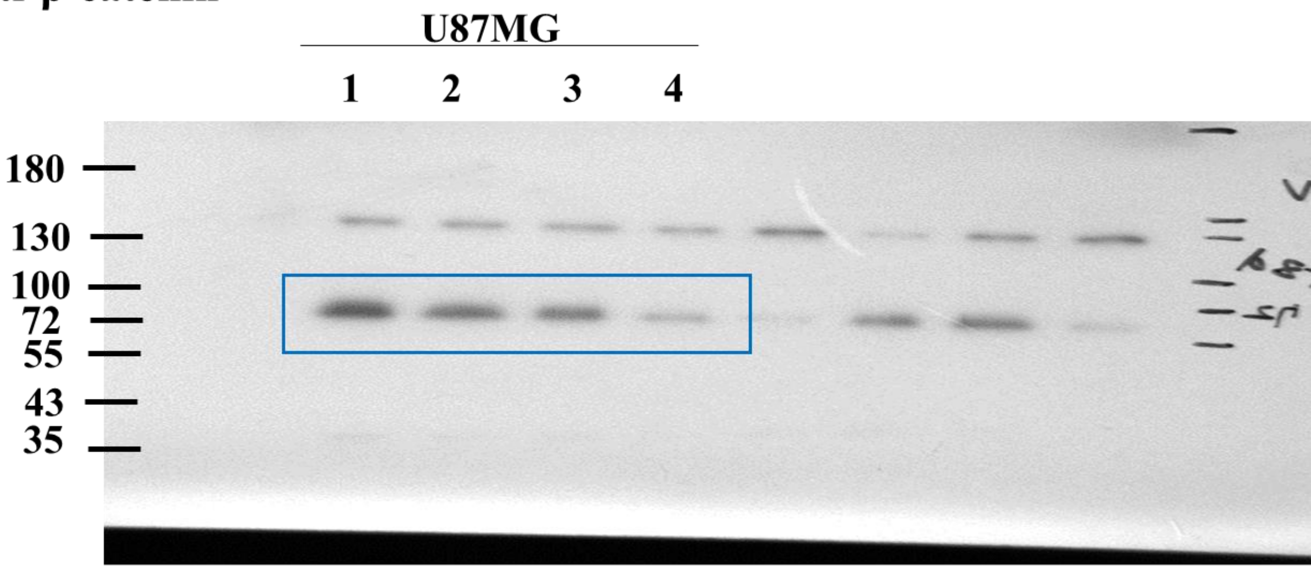

1 : 0.71 : 0.47 : 0.29

Anti- β-catenin

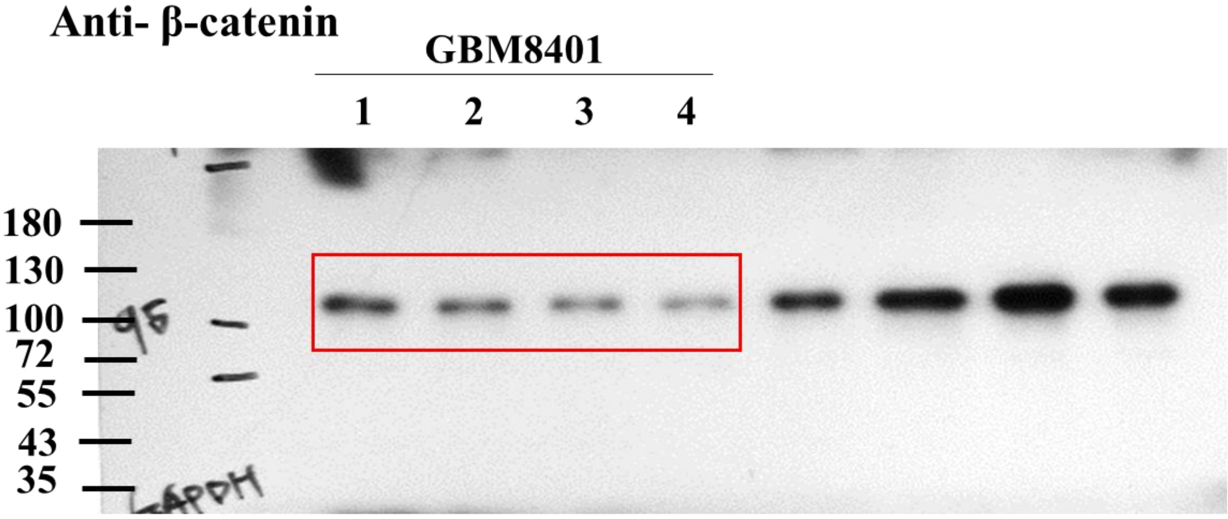

1 : 0.85 : 0.33 : 0.38

Anti-β-catenin (S33/37/41)

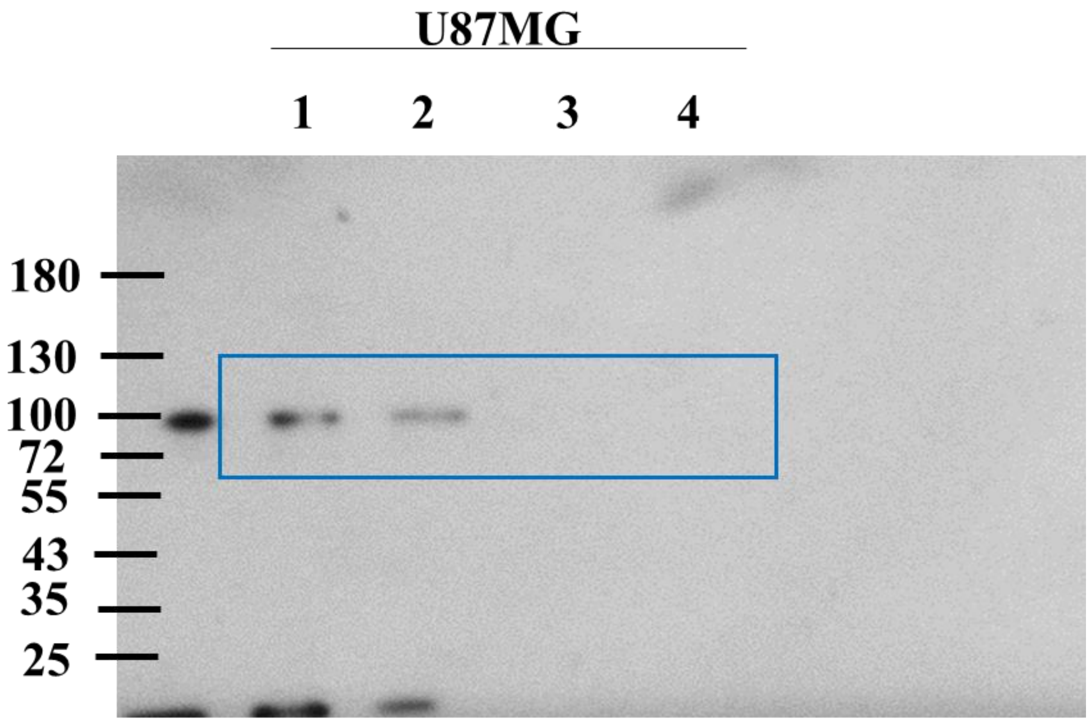

1 : 0.70 : 0.11 : 0.14

Related to Fig.4A-4

Anti-β-catenin (S33/37/41)

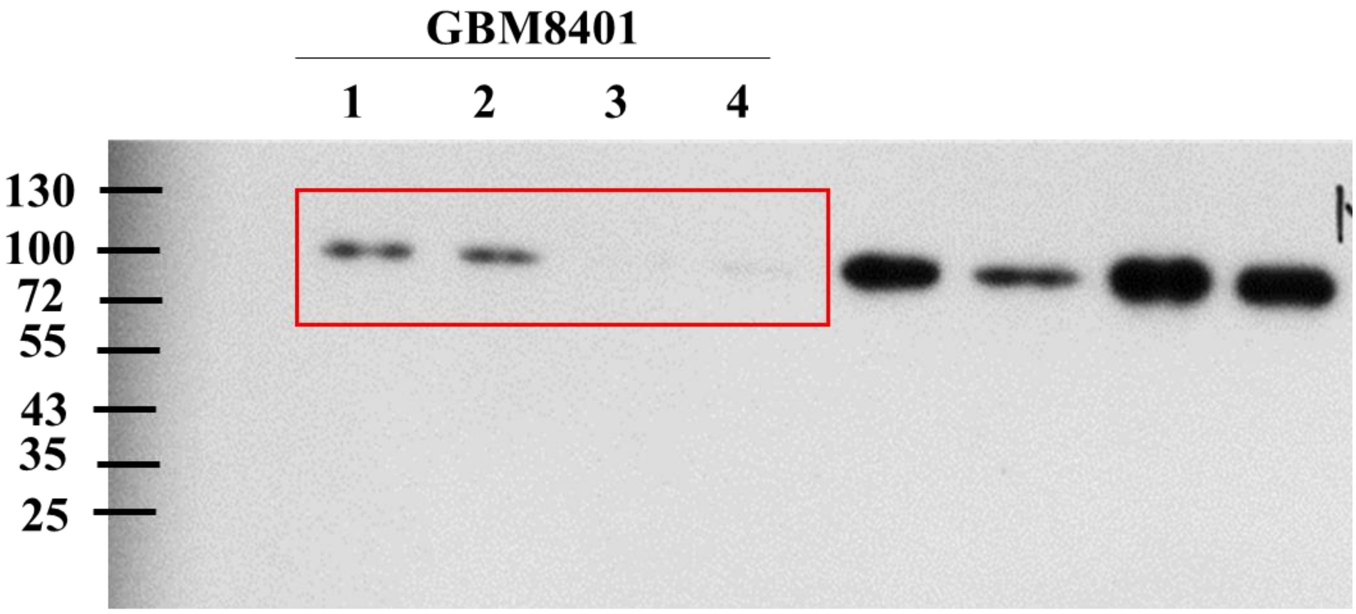

1 : 0.92 : 0.13 : 0.09

Anti-GAPDH

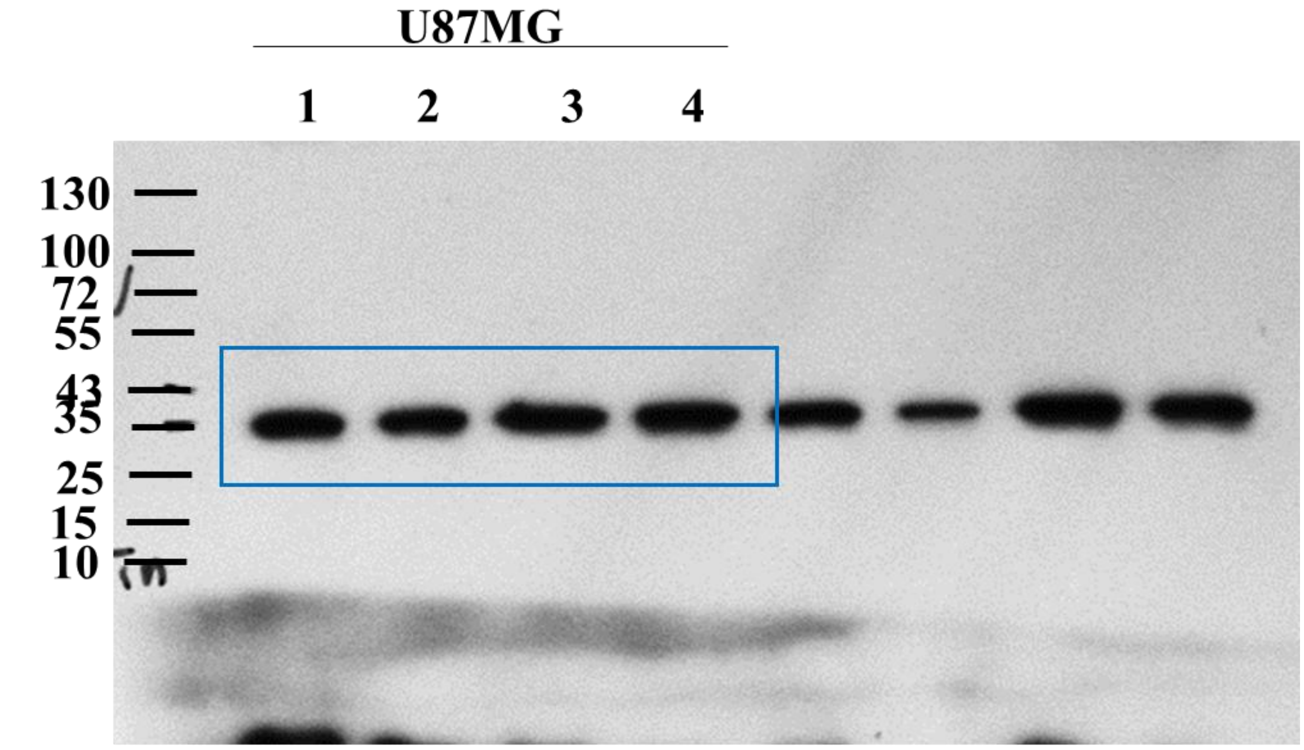

1 : 0.93 : 1.06 : 1.00

Related to Fig.4A-5

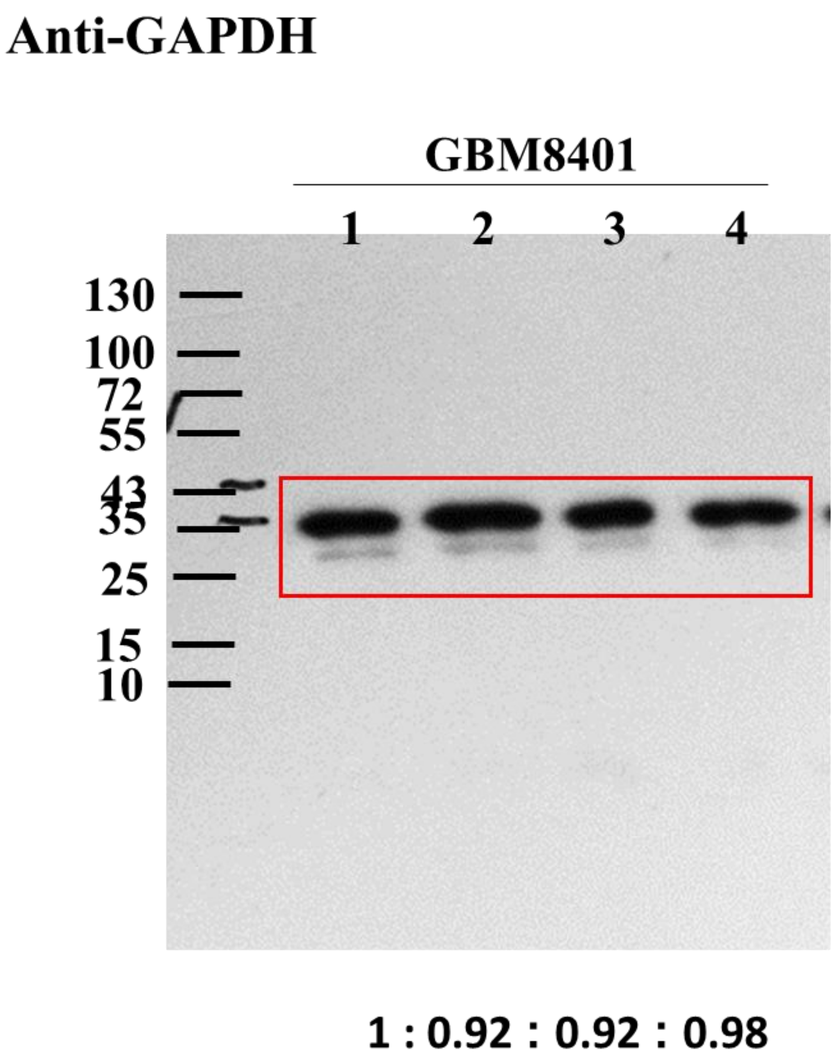

Related to Fig.4B-1

Anti-β-catenin

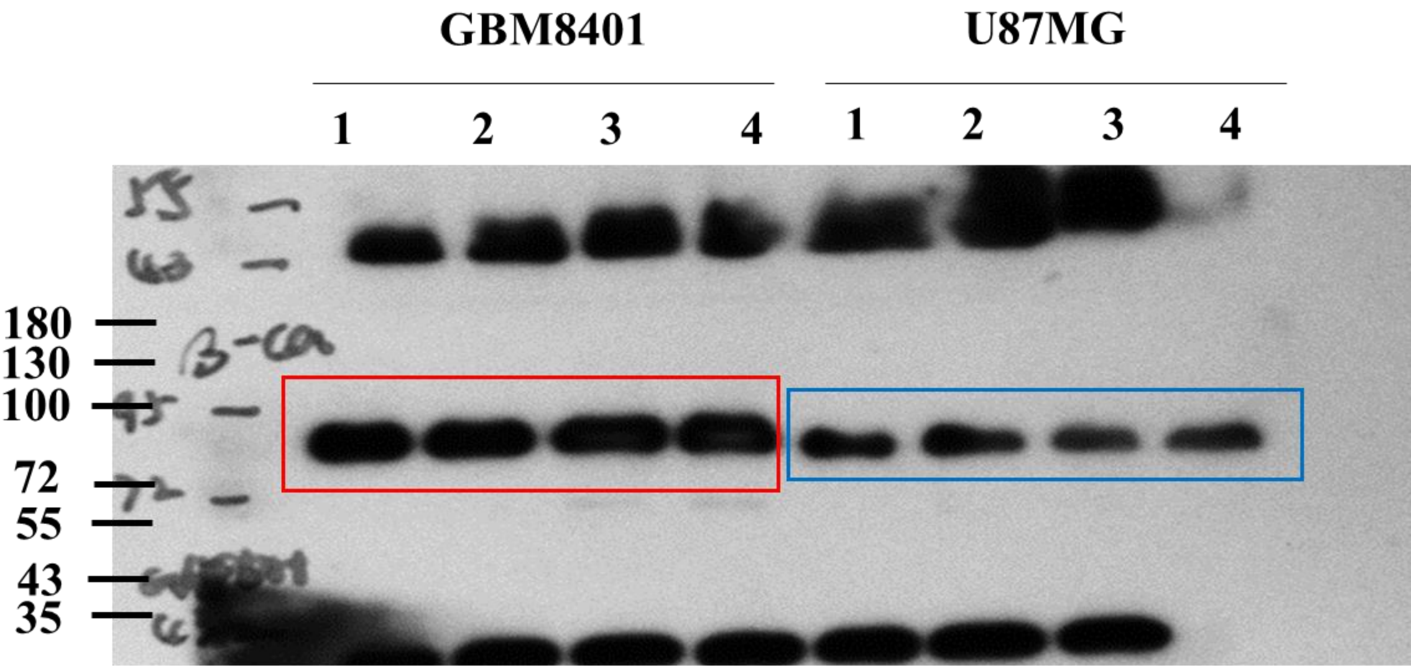

1 : 1.01 : 0.88 : 0.97

1 : 0.89 : 0.78 : 0.76

Anti-β-catenin (S33/37/41)

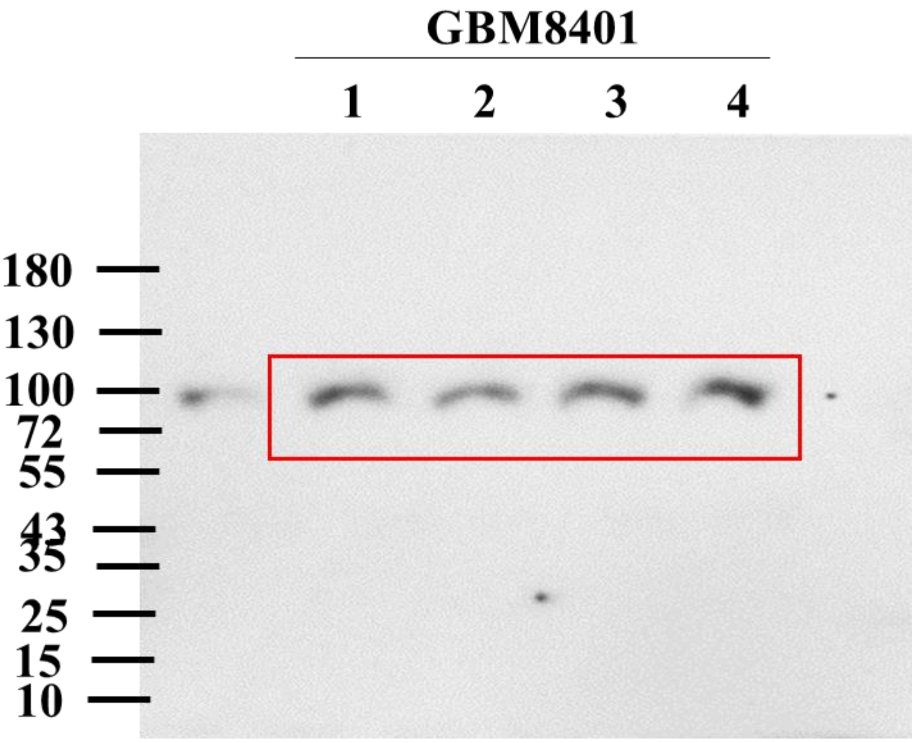

1 : 0.88 : 0.82 : 0.85

Related to Fig.4B-2

Anti-β-catenin (S33/37/41)

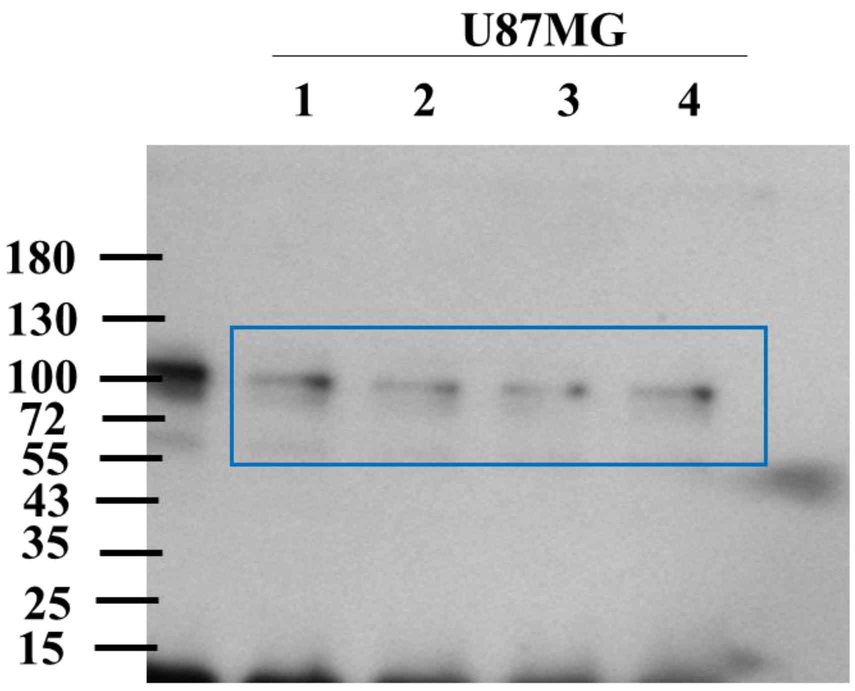

1 : 0.84 : 0.99 : 1.12

Anti-P62

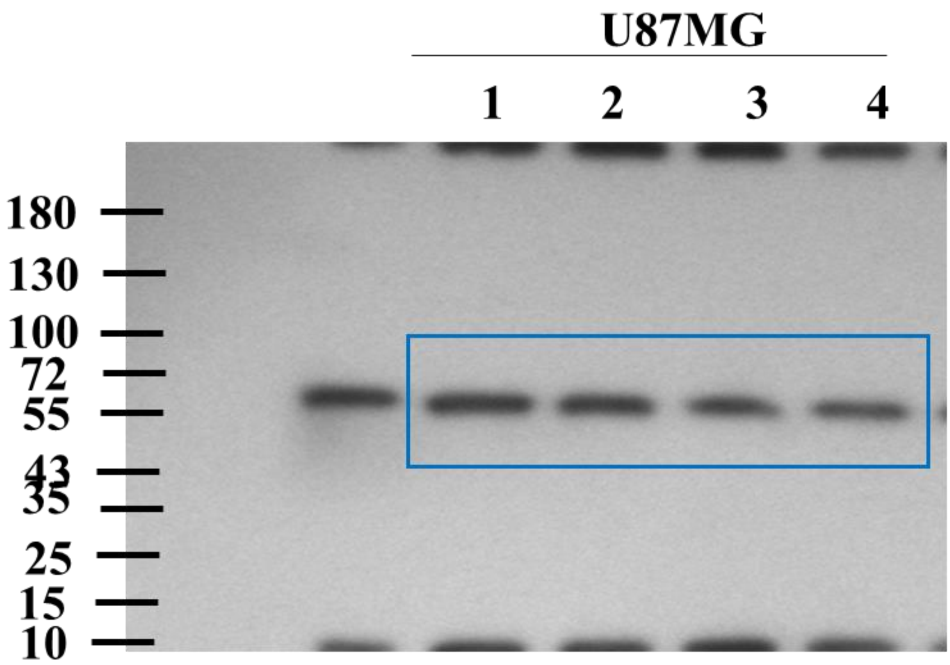

1 : 0.93 : 0.86 : 0.87

Related to Fig.4B-3

Anti-P62

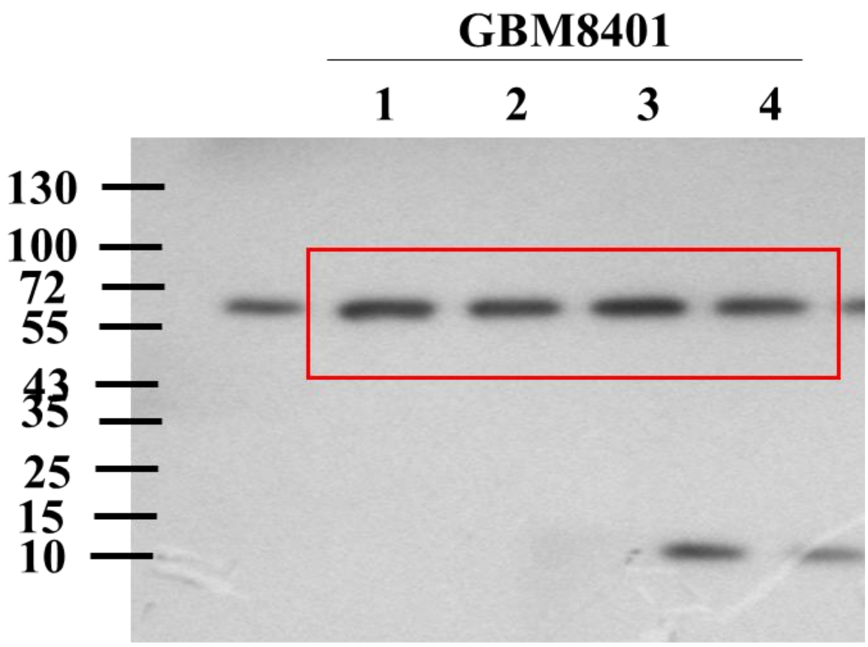

1 : 0.96 : 1.06 : 0.98

Anti-GAPDH

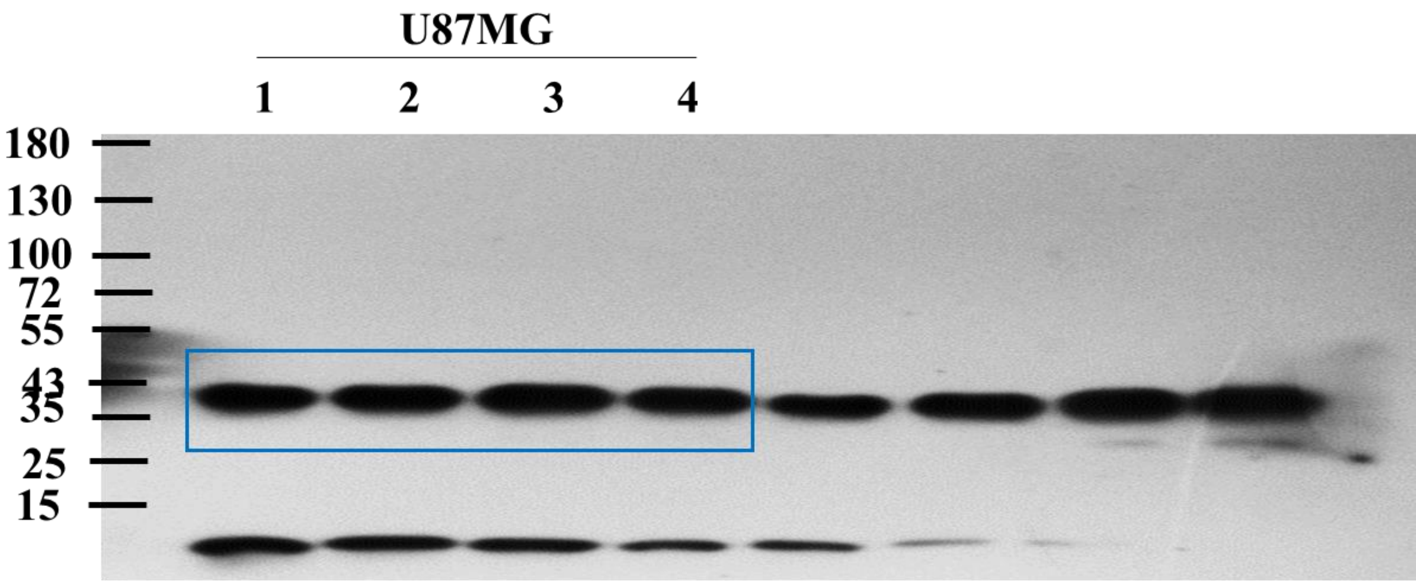

1 : 1.01 : 1.04 : 1.01

Anti- GAPDH

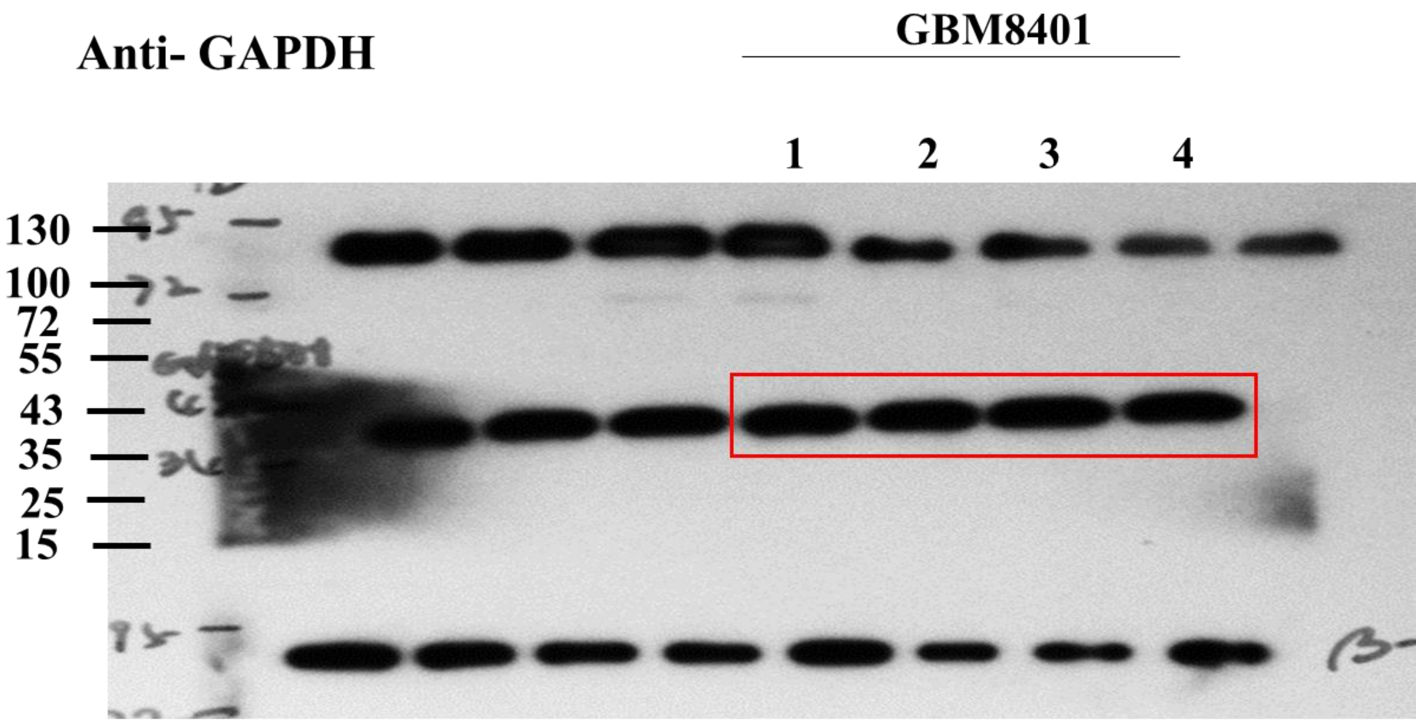

1 : 0.99 : 1.04 : 1.00

Related to Fig.5-1

Anti-P62

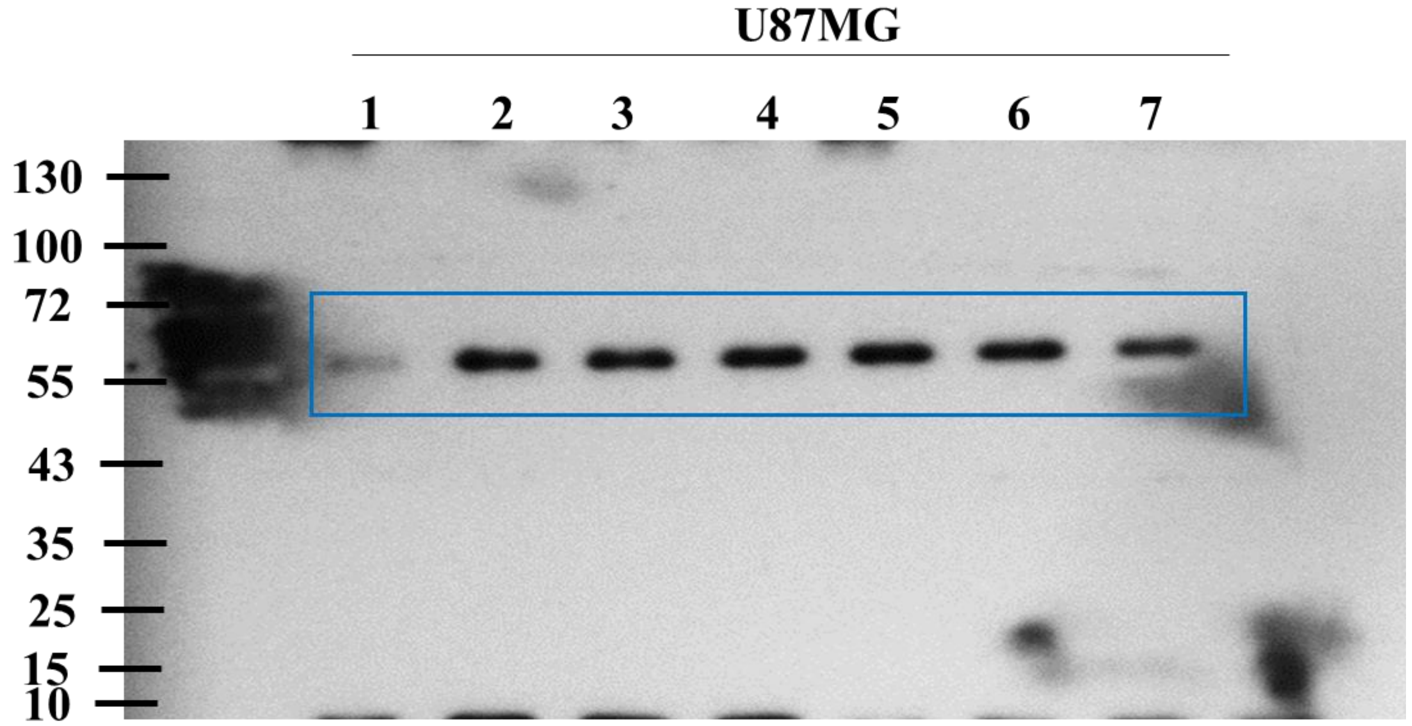

1 : 2.27: 2.33 : 2.16 : 2.14 : 2.06 : 1.52

Anti-LC3I/II

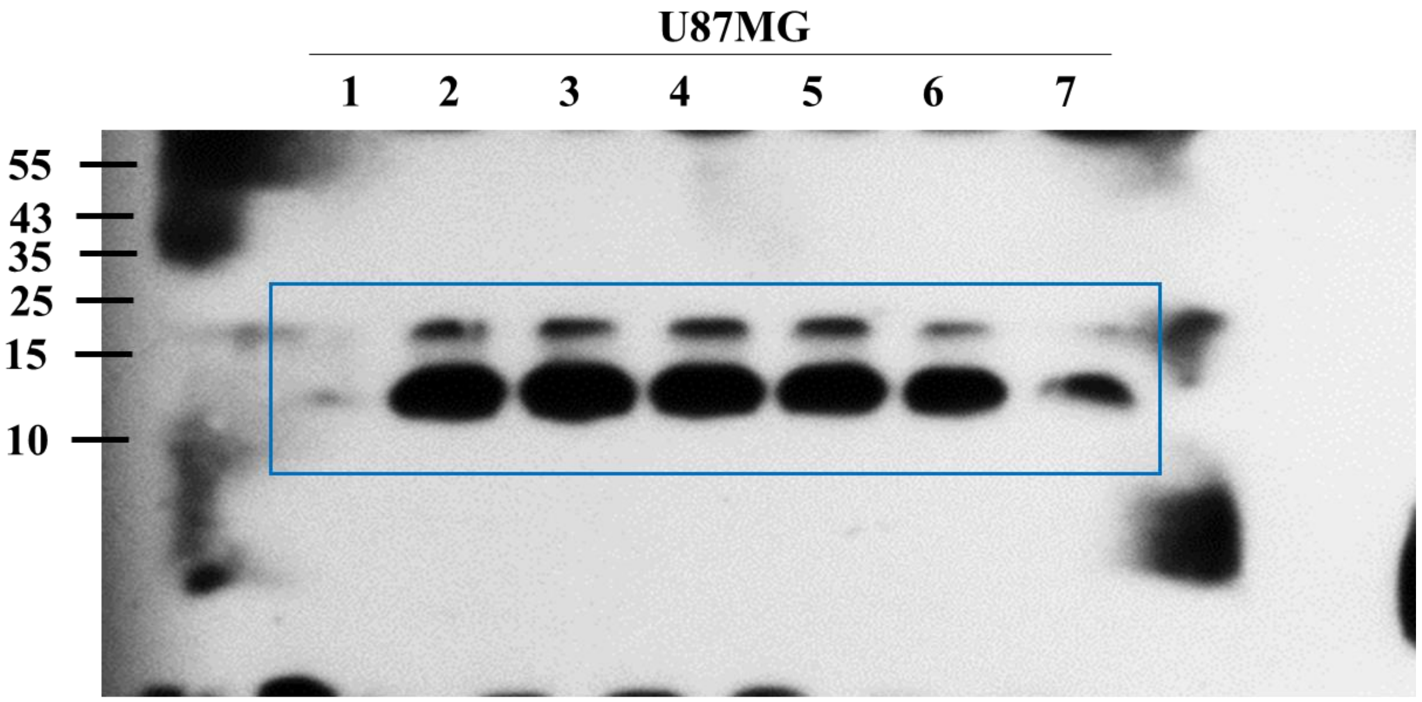

LC3 II **1 : 3.72 : 4.16 : 3.98 : 3.97 : 4.08 : 2.55**

Related to Fig.5-2

Anti-Nestin

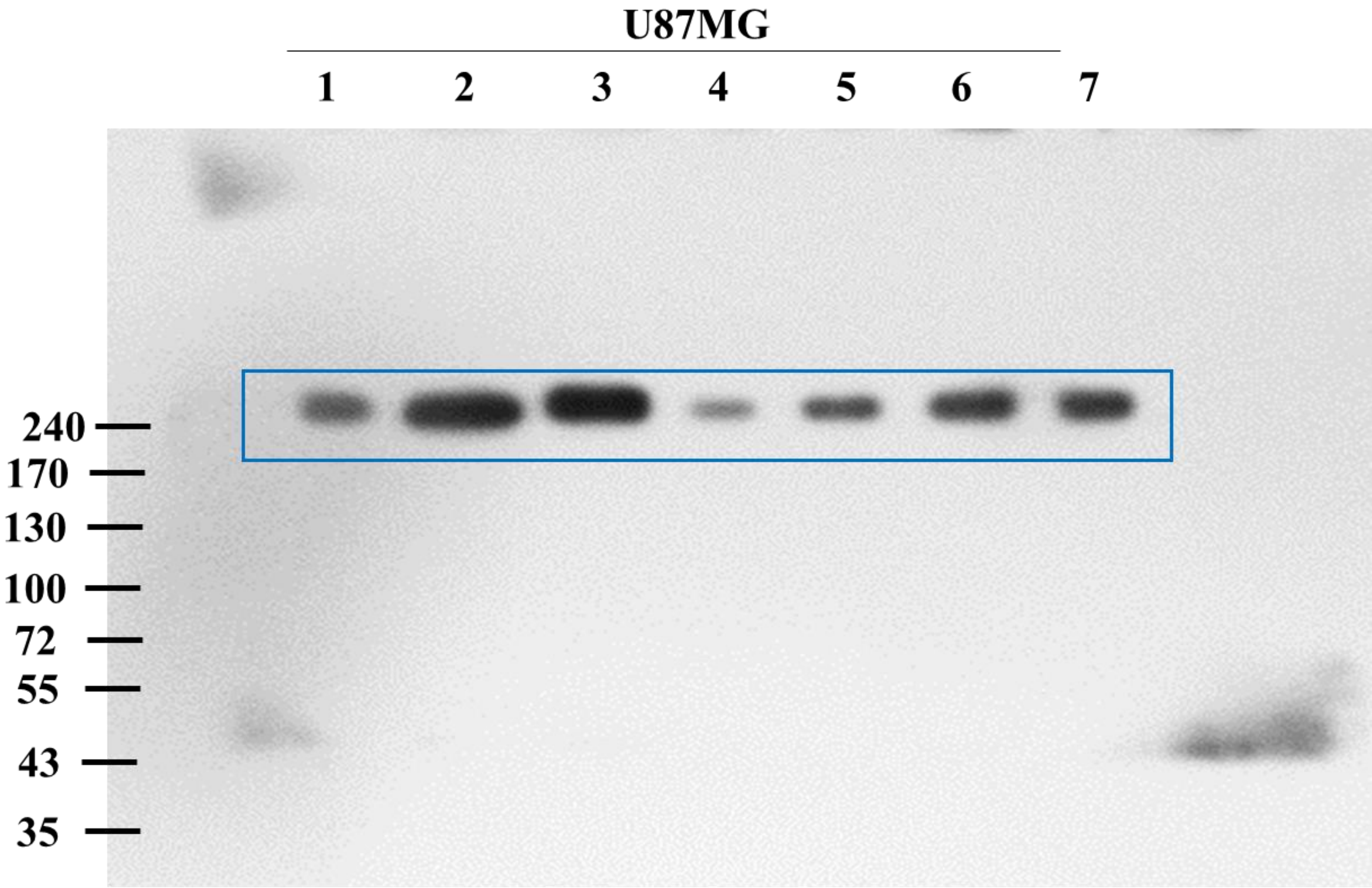

1 : 2.28 : 2.20 : 0.74 : 1.04 : 1.48 : 1.68

Anti-CD44

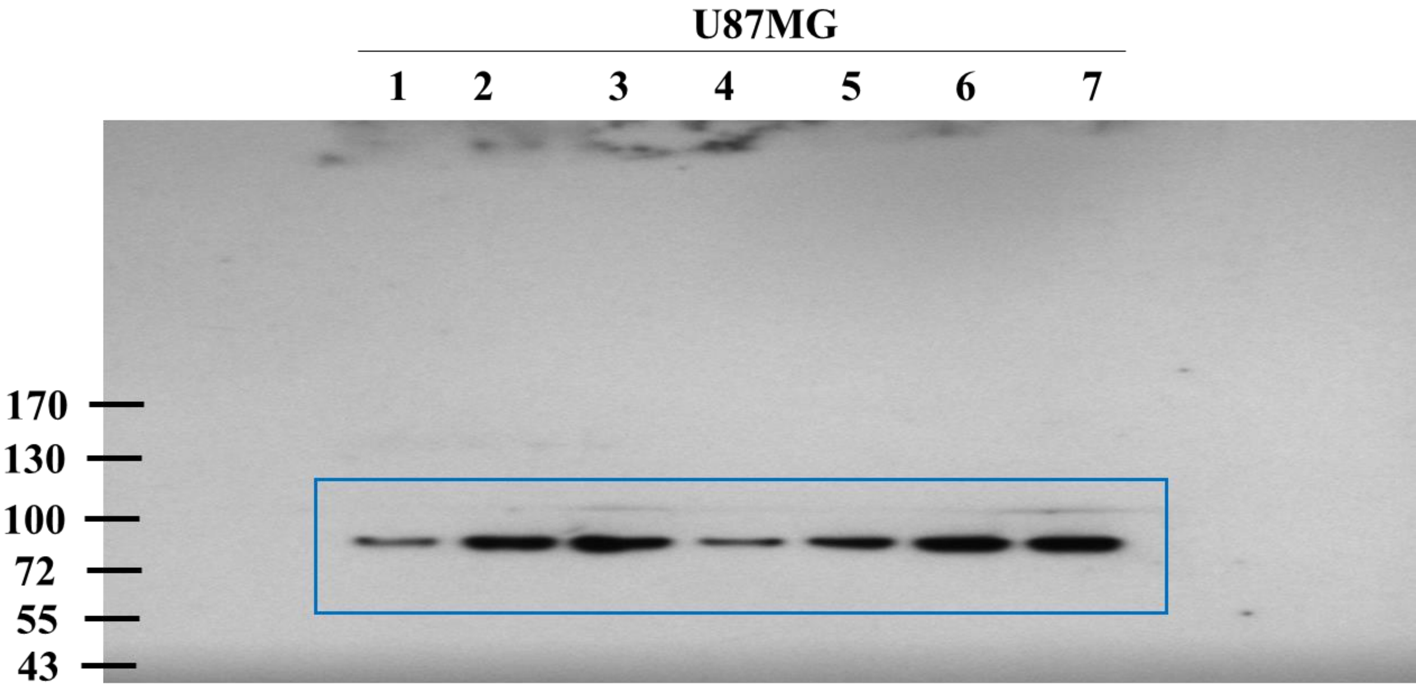

1 : 1.53 : 1.62 : 1.16 : 1.49 : 1.54 : 1.65

Related to Fig.5-3

Anti-CD133

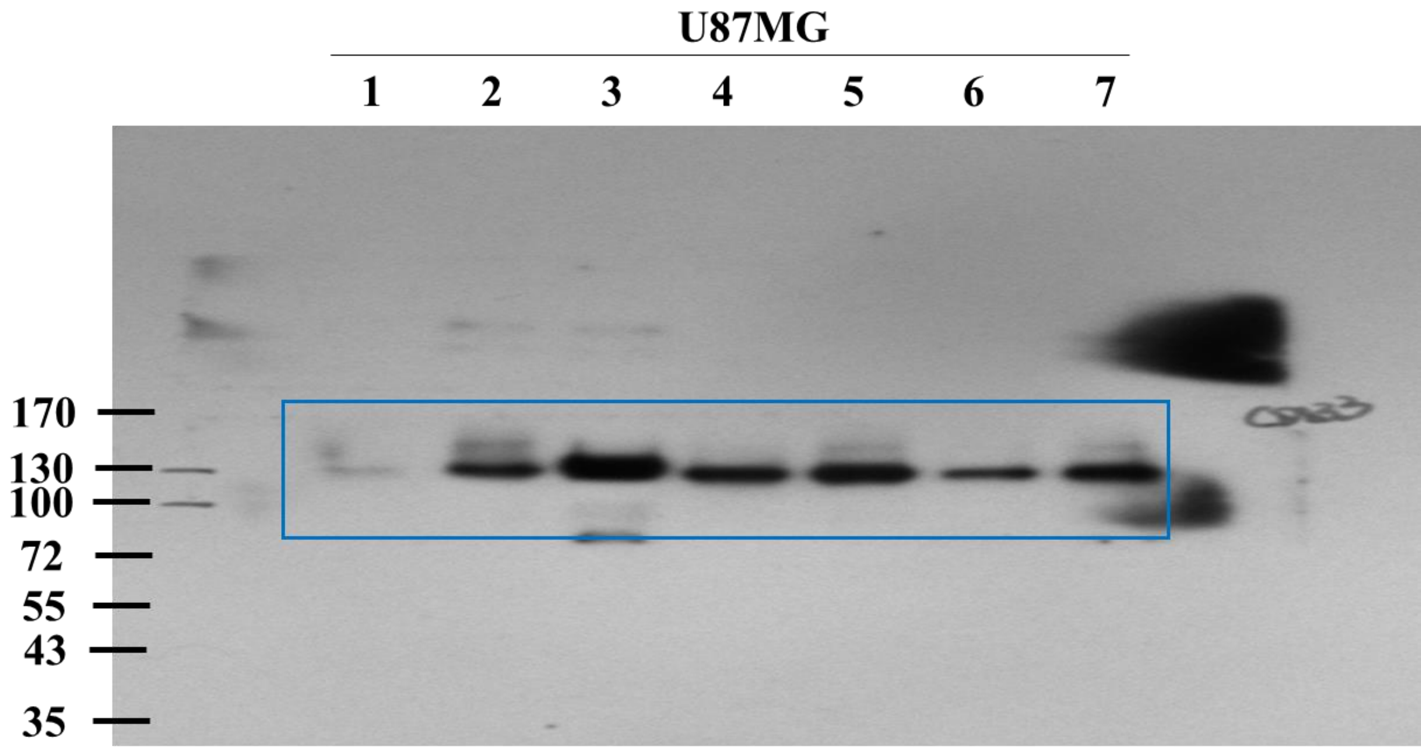

1 : 3.05 : 3.74 : 2.71 : 2.79 : 2.48 : 2.71

Anti-SOX2

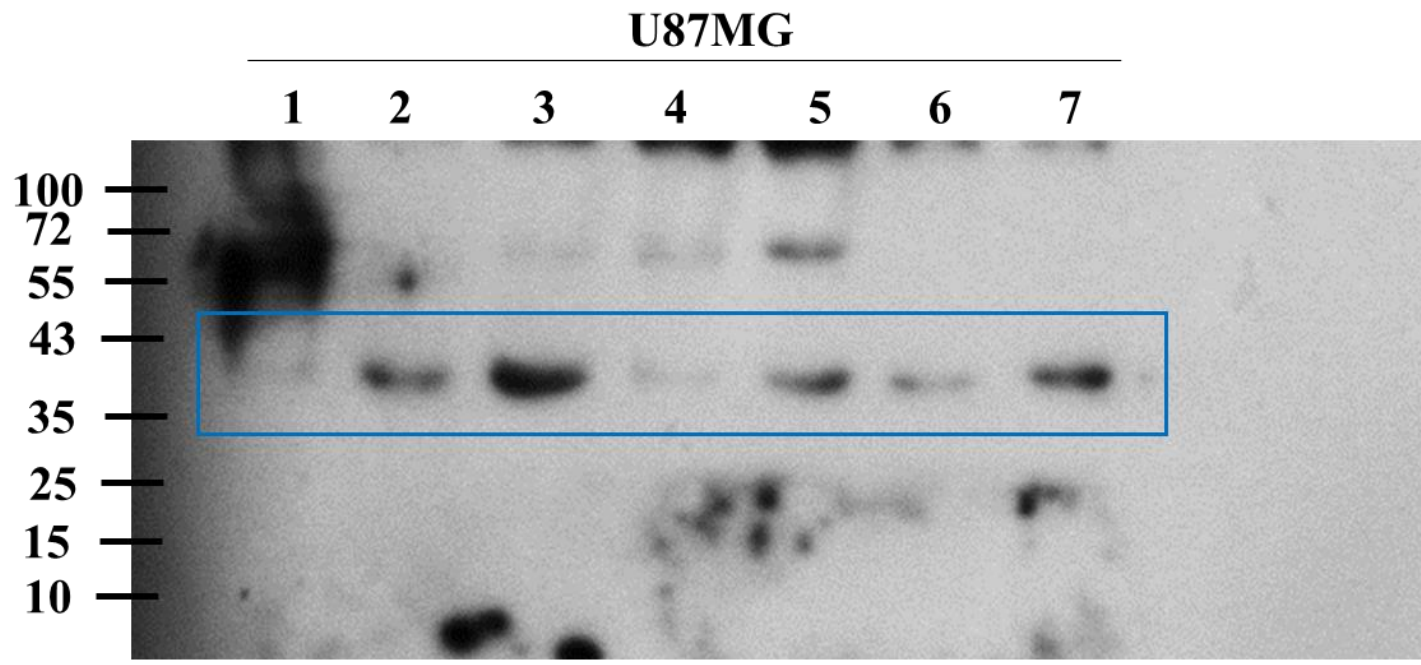

1 : 3.81 : 4.53 : 1.59 : 3.32 : 2.54 : 3.51

Related to Fig.5-4

Anti-GAPDH

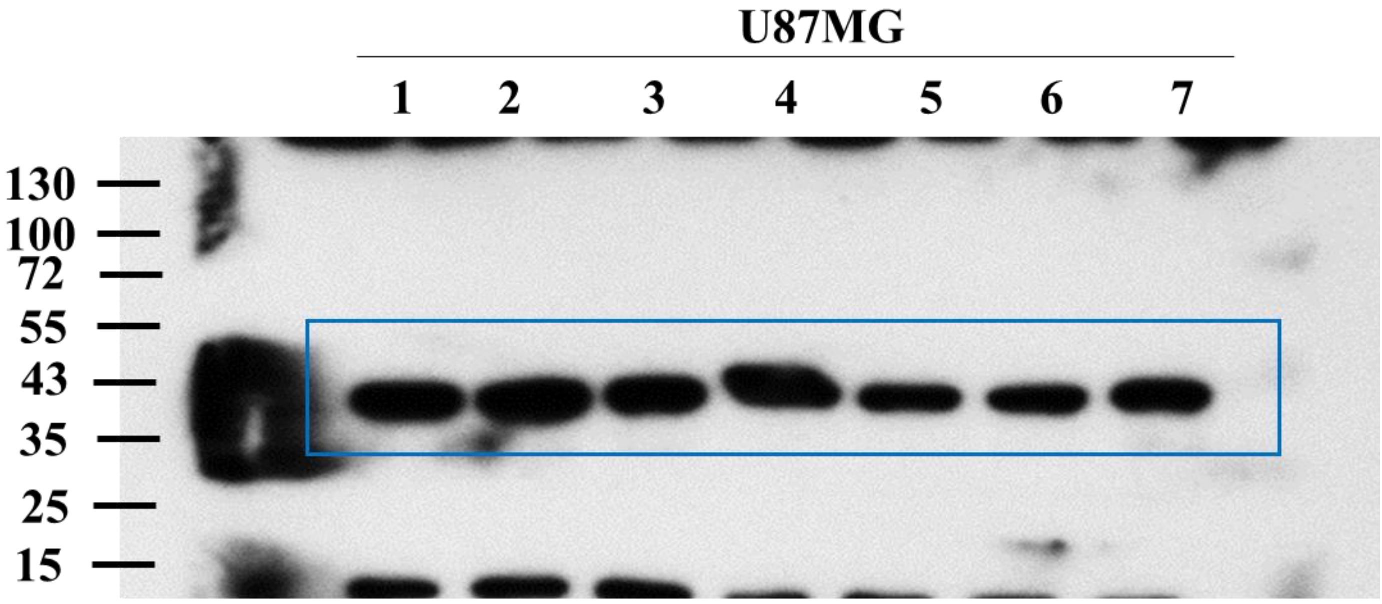

1 : 1 : 0.97 : 0.96 : 0.83 : 0.88 : 1.02

Anti-P62

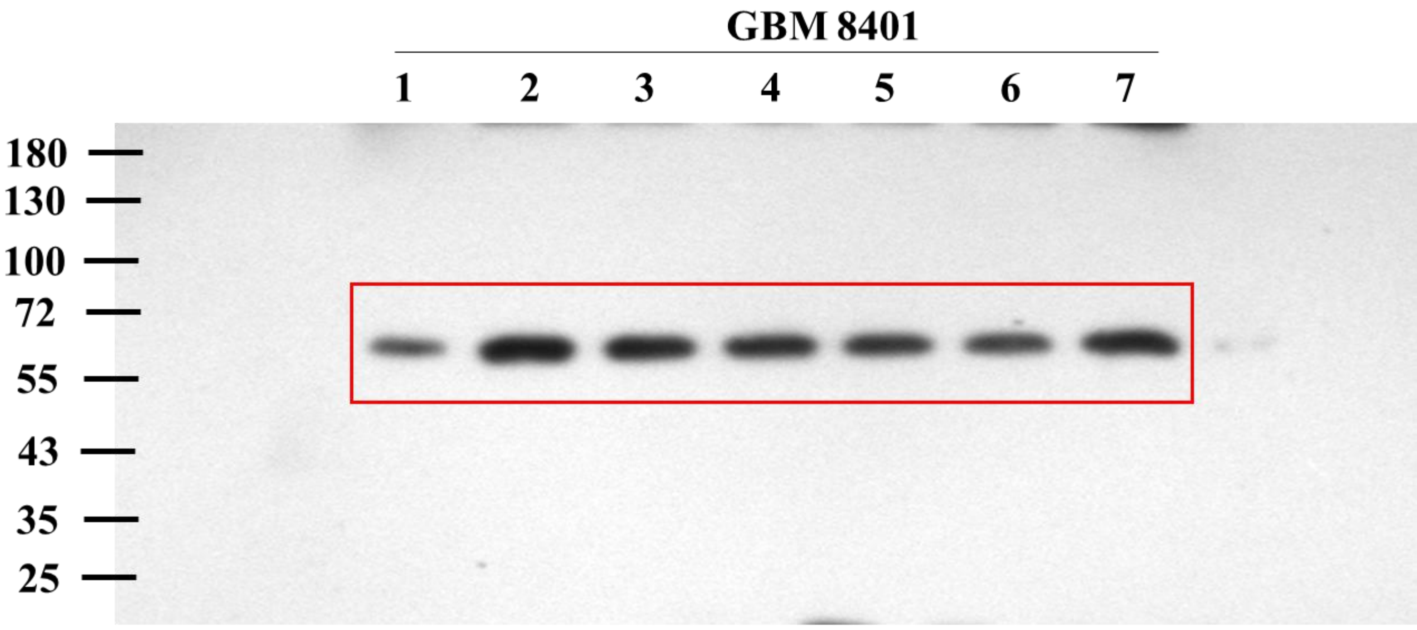

1 : 2.76 : 2.15 : 1.87 : 2.00 : 2.02 : 2.14

Related to Fig.5-5

Anti-LC3I/II

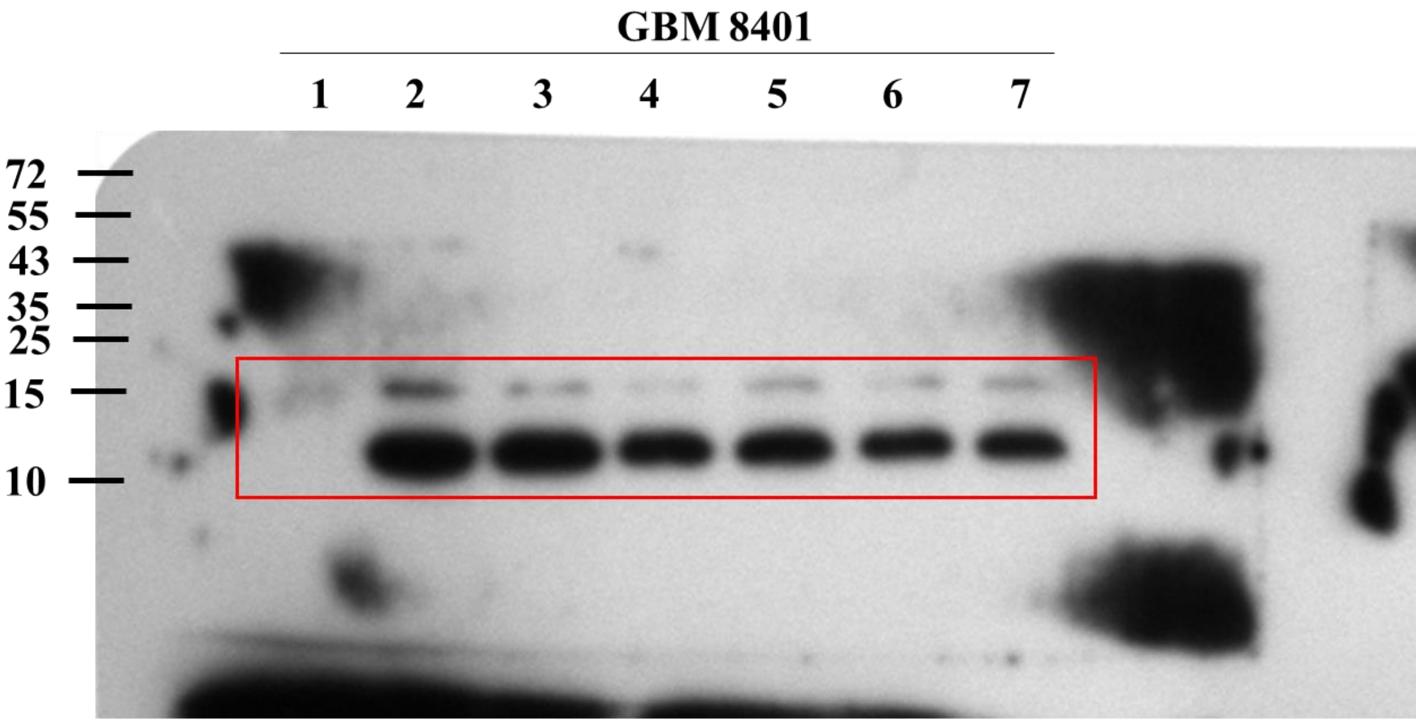

1 : 4.19 : 4.22 : 3.57 : 3.58 : 3.45 : 3.34

Anti-Nestin

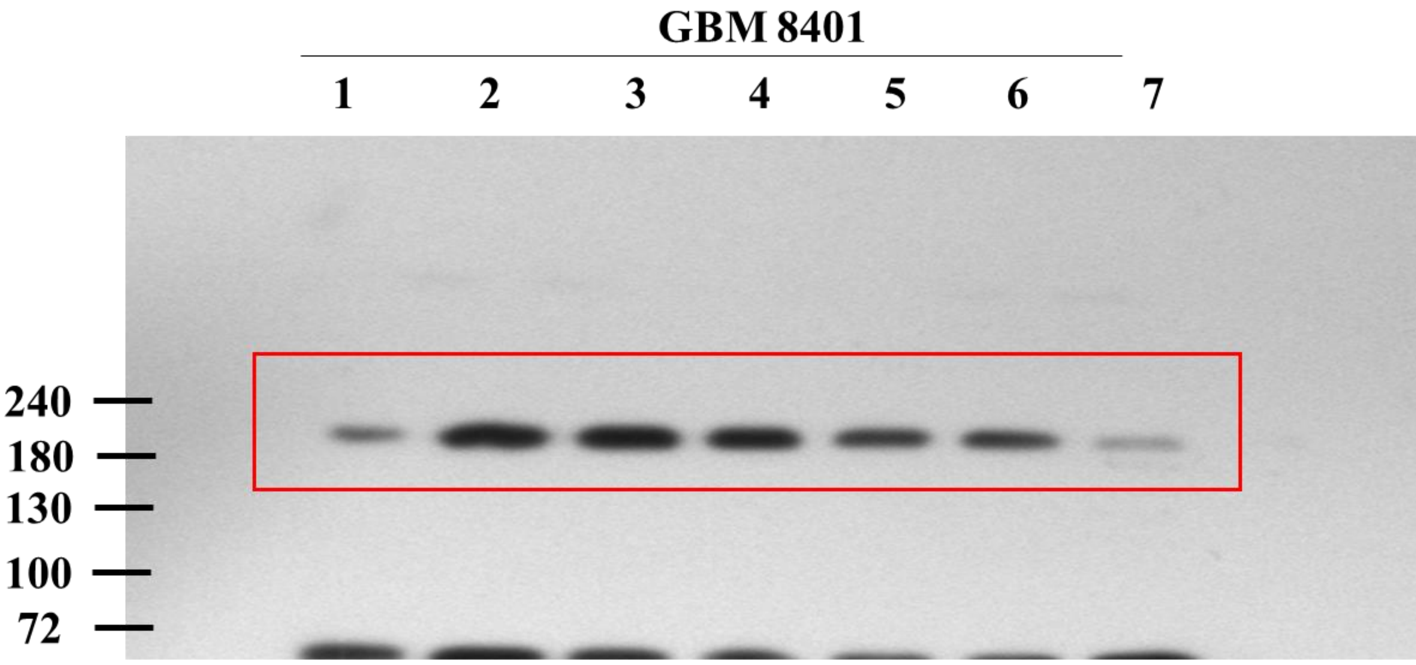

1 : 2.26 : 2.35 : 1.95 : 1.75 : 1.87 : 1.46

Related to Fig.5-6

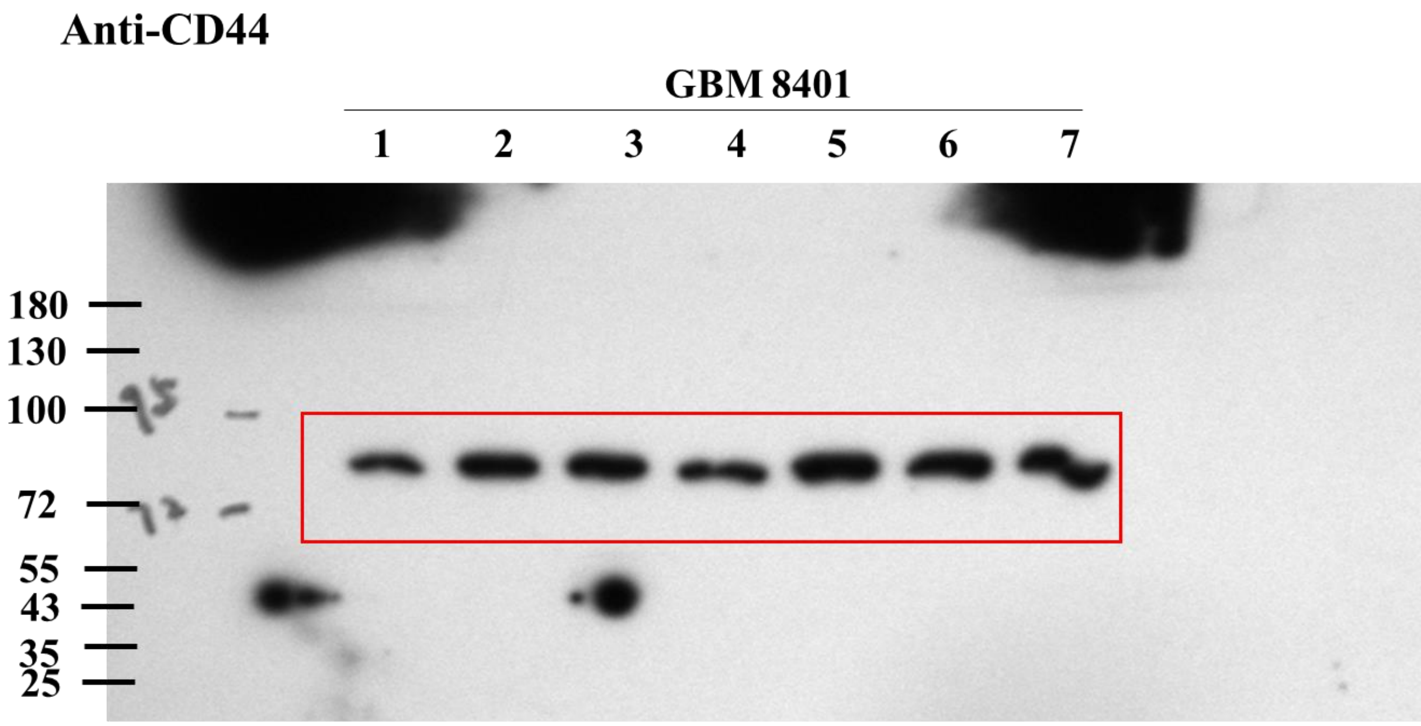

1 : 1.56 : 1.55 : 1.01 : 1.57 : 1.38 : 1.36

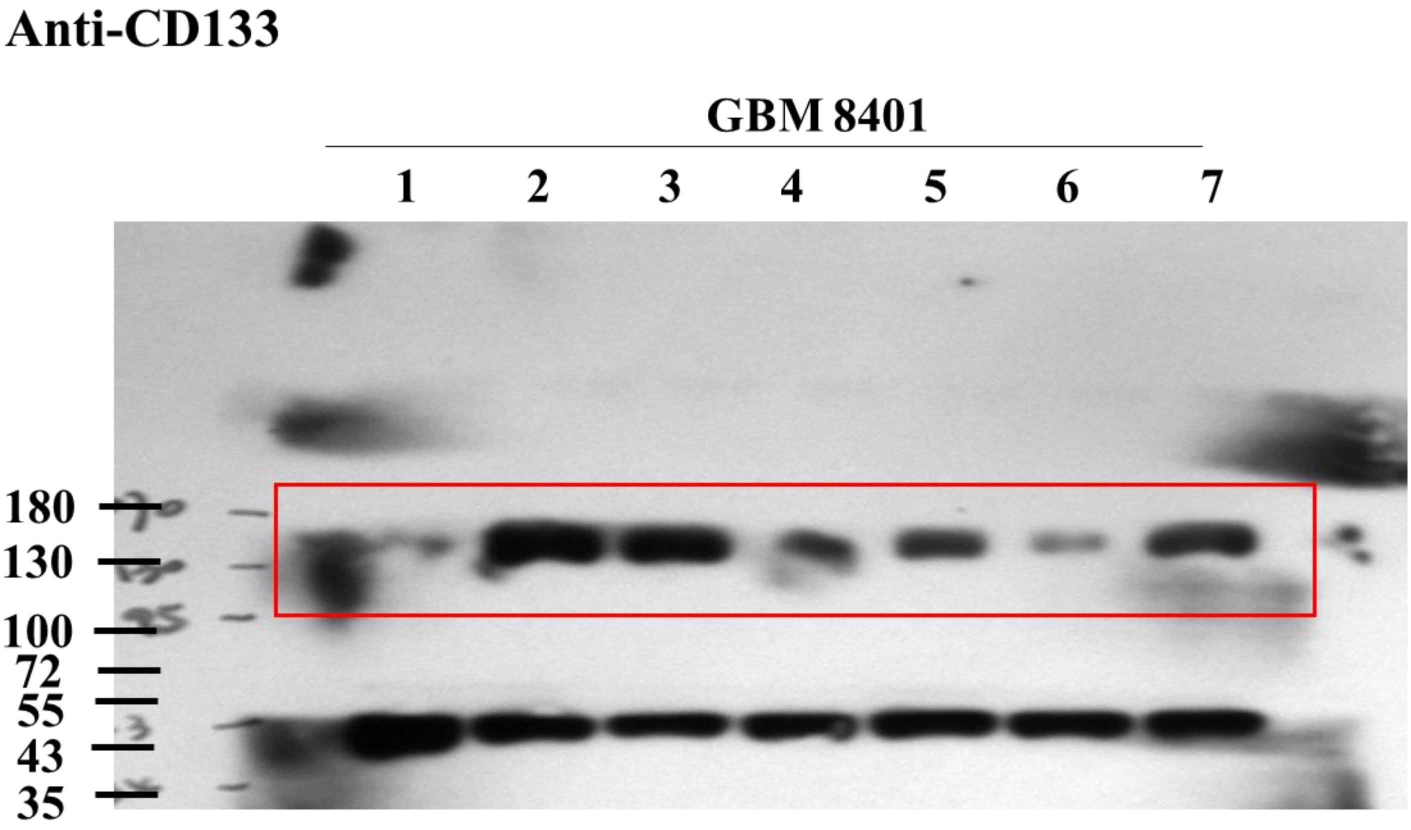

1 : 2.26 : 2.14 : 1.22 : 1.40 : 0.95 : 1.66

Related to Fig.5-7

Anti-SOX2

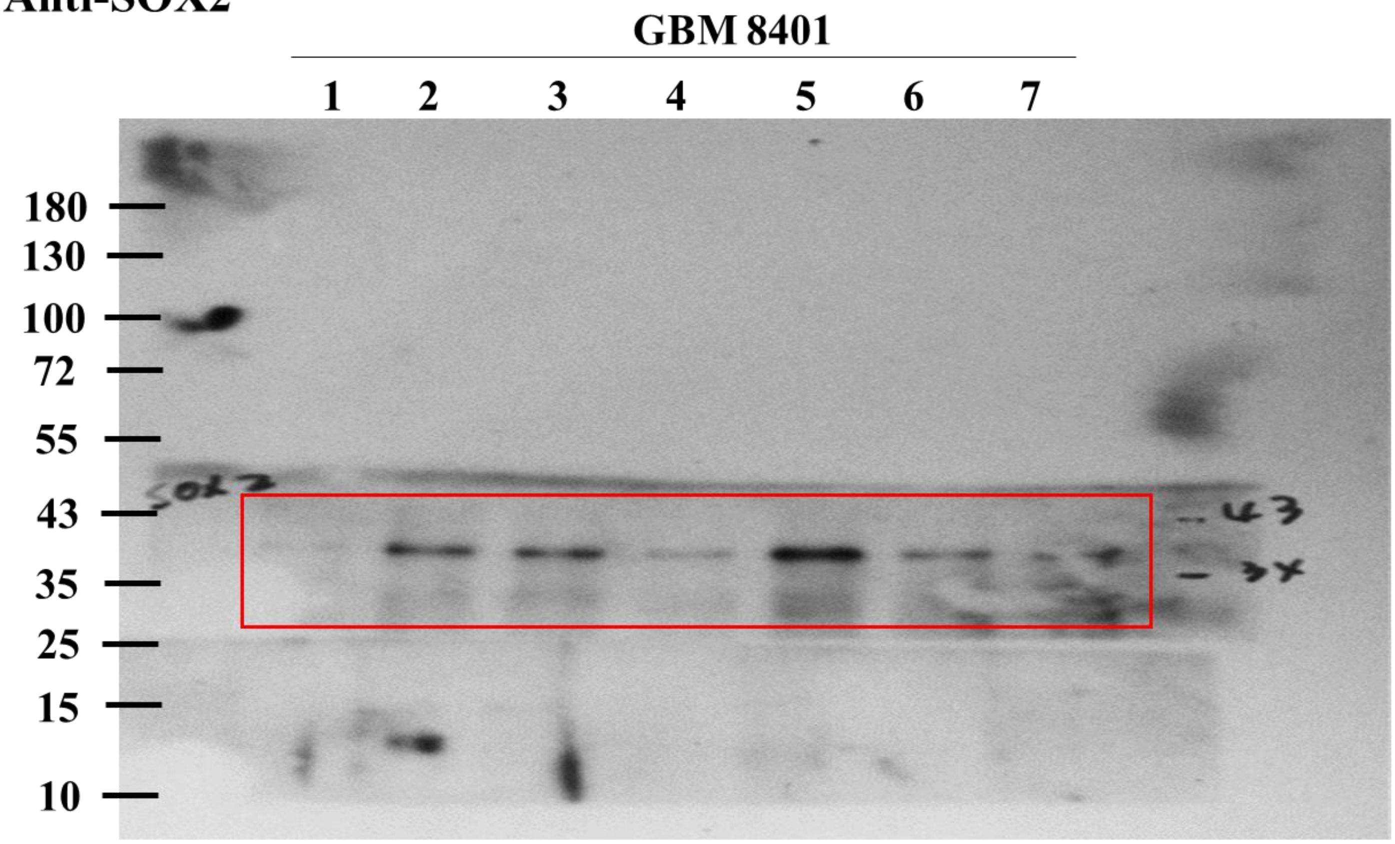

1 : 2.12 : 2.17 : 1.41 : 2.17 : 1.79 : 1.63

Anti-GAPDH

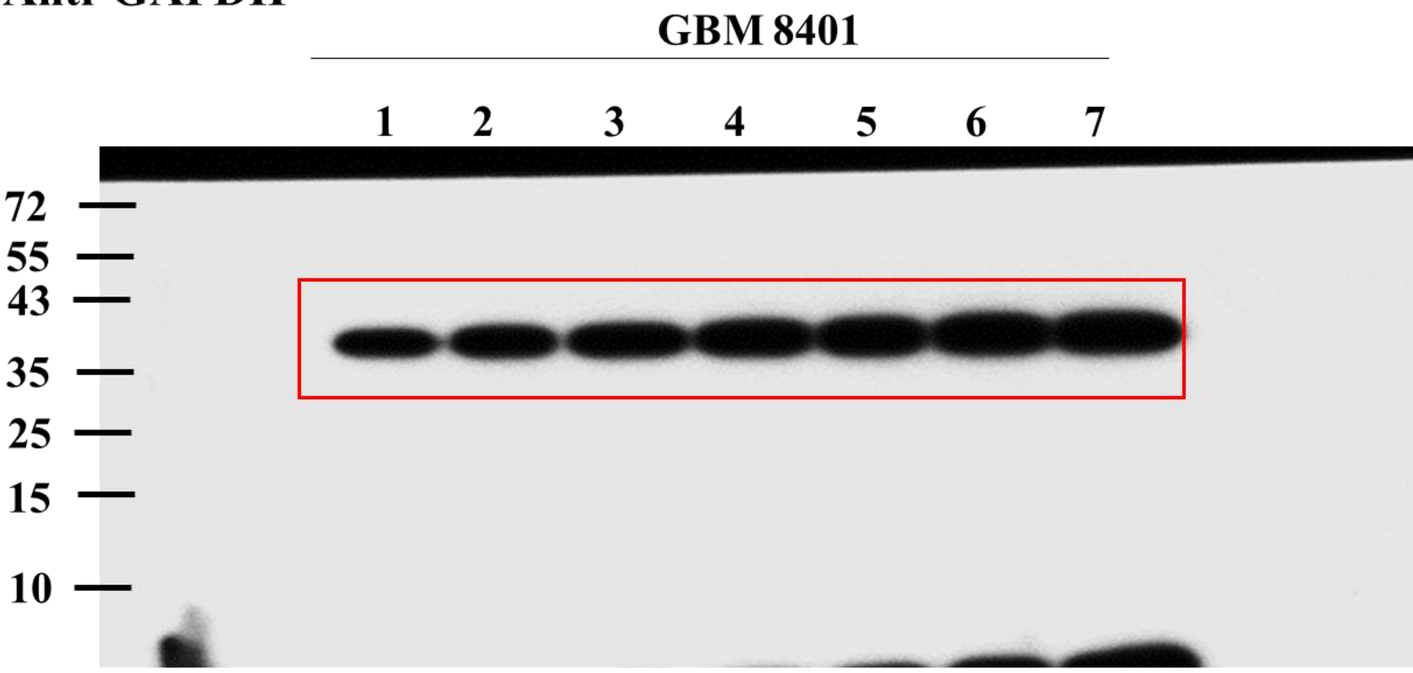

1 : 1.03 : 1.09 : 1.12 : 1.15 : 1.57 : 1.62

Supplement Figure 2-1.

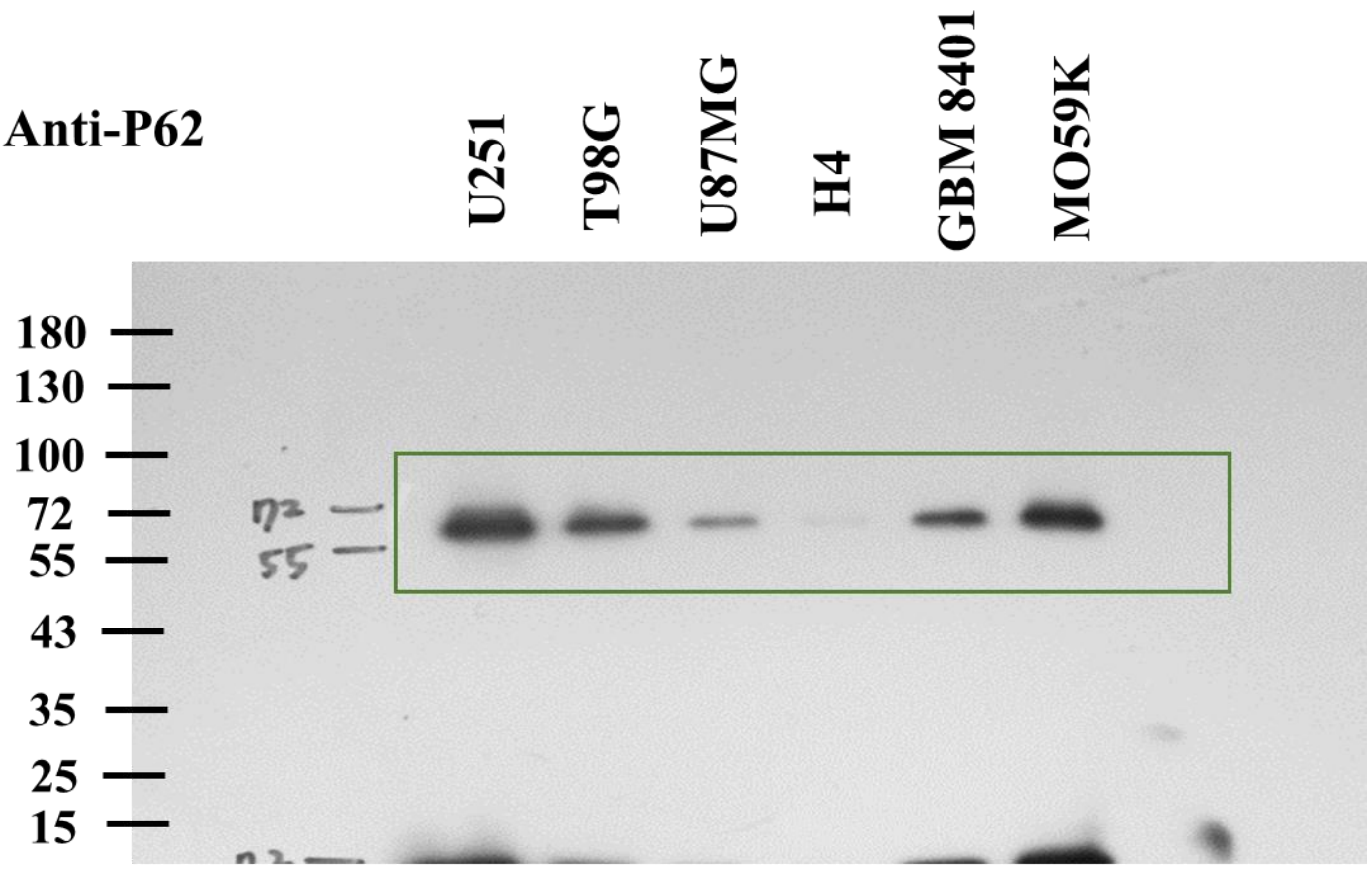

1 : 0.76 : 0.47 : 0.12 : 0.60 : 0.85

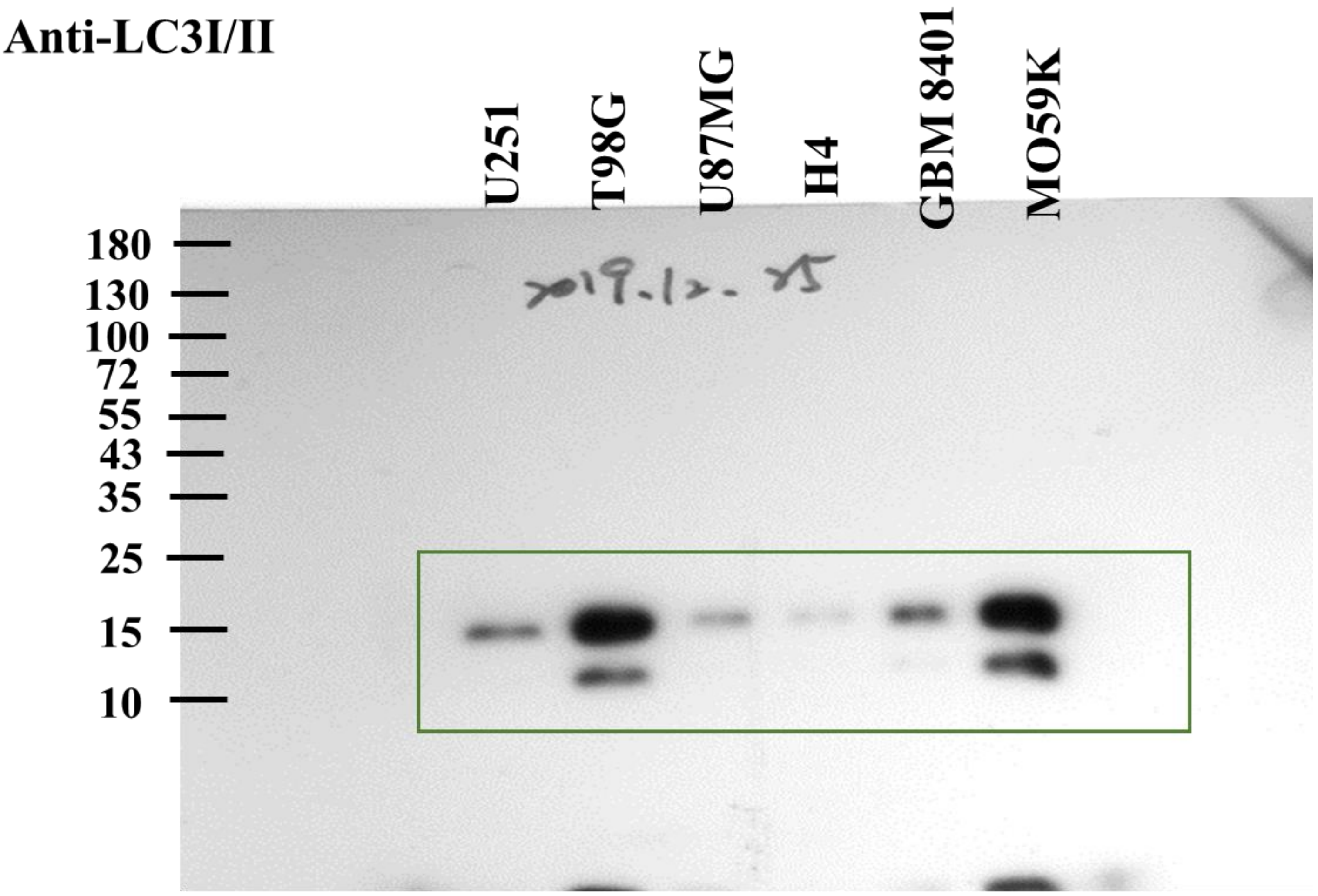

1 : 2.42 : 1.00 : 1.00 : 1.00 : 2.70

Supplement Figure 2-2.

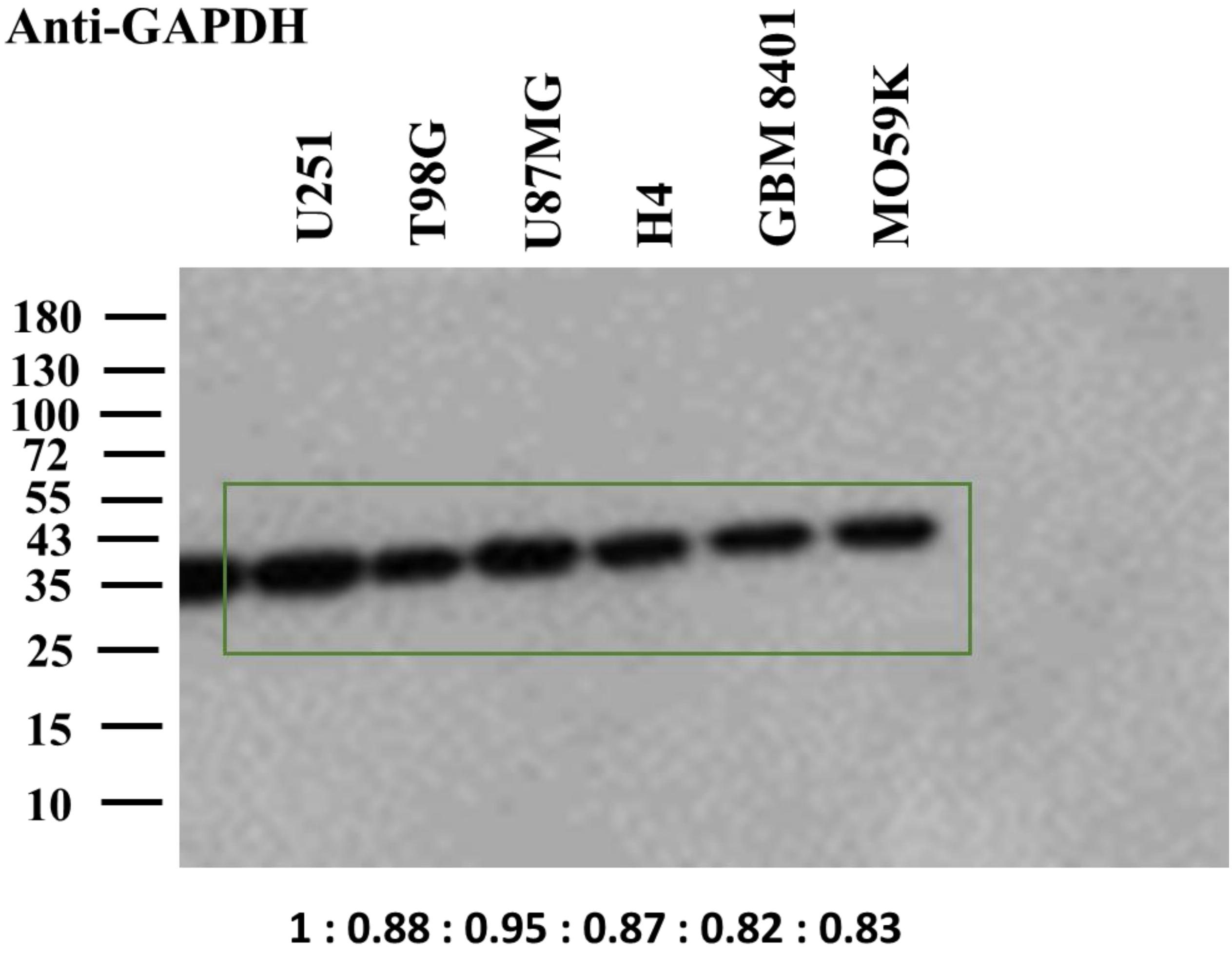

Supplement: Supplementary file 1 [file life-11-00451-s001.zip › life-1197210-supplementary/life-1197210-original western blot figure.pdf]
