# Supplementary material for: Ionizing Radiation Induces Resistant Glioblastoma Stem-Like Cells by Promoting Autophagy via the Wnt/β-Catenin Pathway
Source: Life (Basel). 2021 May 18;11(5):451. doi: 10.3390/life11050451 (PMC8157563; doi:10.3390/life11050451)
Supplement: Supplementary file 1 [file life-11-00451-s001.zip › life-1197210-supplementary/life-1197210-supplementary.pdf]

life

MDPI

Article

Ionizing Radiation Induces Resistant Glioblastoma Stem-Like Cells by Promoting Autophagy via the Wnt/β-Catenin Pathway

Cheng-Yu Tsai [1,2], Huey-Jiun Ko [3,4], Chi-Ying F. Huang [5], Ching-Yi Lin [2], Shean-Jaw Chiou [4], Yu-Feng Su [2], Ann-Shung Lieu [2], Joon-Khim Loh [2,3], Aij-Lie Kwan [1,2,3,*], Tsung-Hsien Chuang [1,6,*] and Yi-Ren Hong [1,3,4,7,8,*]

1 Environmental and Occupational Medicine, College of Medicine, Kaohsiung Medical University, Kaohsiung 807, Taiwan; 1030459@kmuh.org.tw
2 Department of Neurosurgery, Kaohsiung Medical University Hospital, Kaohsiung 807, Taiwan; 4a1h0010@gmail.com (C.-Y.L.); 870082@kmuh.org.tw (Y.-F.S.); 770223@kmuh.org.tw (A.-S.L.); jokhlo@kmu.edu.tw (J.-K.L.)
3 Graduate Institute of Medicine, College of Medicine, Kaohsiung Medical University, Kaohsiung 807, Taiwan; o870391@yahoo.com.tw (H.-J.K.)
4 Department of Biochemistry, College of Medicine, Kaohsiung Medical University, Kaohsiung 807, Taiwan; sheanjaw@kmu.edu.tw
5 Department of Biotechnology and Laboratory Science in Medicine, Institute of Biopharmaceutical Sciences, National Yang-Ming University, Taipei 112, Taiwan; cyhuang5@ym.edu.tw
6 Immunology Research Center, National Health Research Institutes, Miaoli 35053, Taiwan
7 Department of Biological Sciences, National Sun Yat-Sen University, Kaohsiung 804, Taiwan
8 Department of Medical Research, Kaohsiung Medical University Hospital, Kaohsiung 807, Taiwan
* Correspondence: ailikw@kmu.edu.tw (A.-L.K.); thchuang@nhri.edu.tw (T.-H.C.); m835016@kmu.edu.tw (Y.-R.H.); Tel.: +886-37-206166-37611 (A.-L.K.); +886-7-3121101-5386 (T.-H.C.); +886-7-3121101-5880 (Y.-R.H.); Fax: +886-37-586642 (A.-L.K.); +886-7-3218309 (T.-H.C.); +886-07-3215039 (Y.-R.H.)

Abstract: Therapeutic resistance in recurrent glioblastoma multiforme (GBM) after concurrent chemoradiotherapy (CCRT) is a challenging issue. Although standard fractionated radiation is essential to treat GBM, it has led to local recurrence along with therapy-resistant cells in the ionizing radiation (IR) field. Lines of evidence showed cancer stem cells (CSCs) play a vital role in therapy resistance in many cancer types, including GBM. However, the molecular mechanism is poorly understood. Here, we proposed that autophagy could be involved in GSC induction for radioresistance. In a clinical setting, patients who received radiation/chemotherapy had higher LC3II expression and showed poor overall survival compared with those with low LC3 II. In a cell model, U87MG and GBM8401 expressed high level of stemness markers CD133/CD44/Nestin, and autophagy marker P62/LC3II after receiving standard fractionated IR. Furthermore, Wnt/β-catenin proved to be a potential pathway and related to P62 by using proteasome inhibitor (MG132). Moreover, pharmacological inhibition of autophagy with BAF and CQ inhibit GSC cell growth by impairing autophagy flux as demonstrated by decrease Nestin, CD133, and SOX-2 levels. In conclusion, we demonstrated that fractionated IR could induce GSCs with the stemness phenotype by P62-mediated autophagy through the Wnt/β-catenin for radioresistance. This study offers a new therapeutic strategy for targeting GBM in the future.

Keywords: GBM; CSC; ionizing radiation (IR), GSC; Wnt/β-Catenin; autophagy; radiation resistance

Citation: Tsai, C.-Y.; Ko, H.-J.; Huang, C.-Y.F.; Lin, C.-Y.; Chiou, S.-J.; Su, Y.-F.; Lieu, A.-S.; Loh, J.-K.; Kwan, A.-L.; Chuang, T.-H.; et al. Ionizing Radiation Induces Resistant Glioblastoma Stem-Like Cells by Promoting Autophagy via the Wnt/β-Catenin Pathway. *Life* **2021**, *11*, 451. https://doi.org/10.3390/life11050451

Academic Editor: Andrew Edet Ekpenyong

Received: 9 April 2021
Accepted: 17 May 2021
Published: 18 May 2021

Publisher's Note: MDPI stays neutral with regard to jurisdictional claims in published maps and institutional affiliations.

Copyright: © 2021 by the authors. Licensee MDPI, Basel, Switzerland. This article is an open access article distributed under the terms and conditions of the Creative Commons Attribution (CC BY) license (http://creativecommons.org/licenses/by/4.0/).

Supplementary Materials:

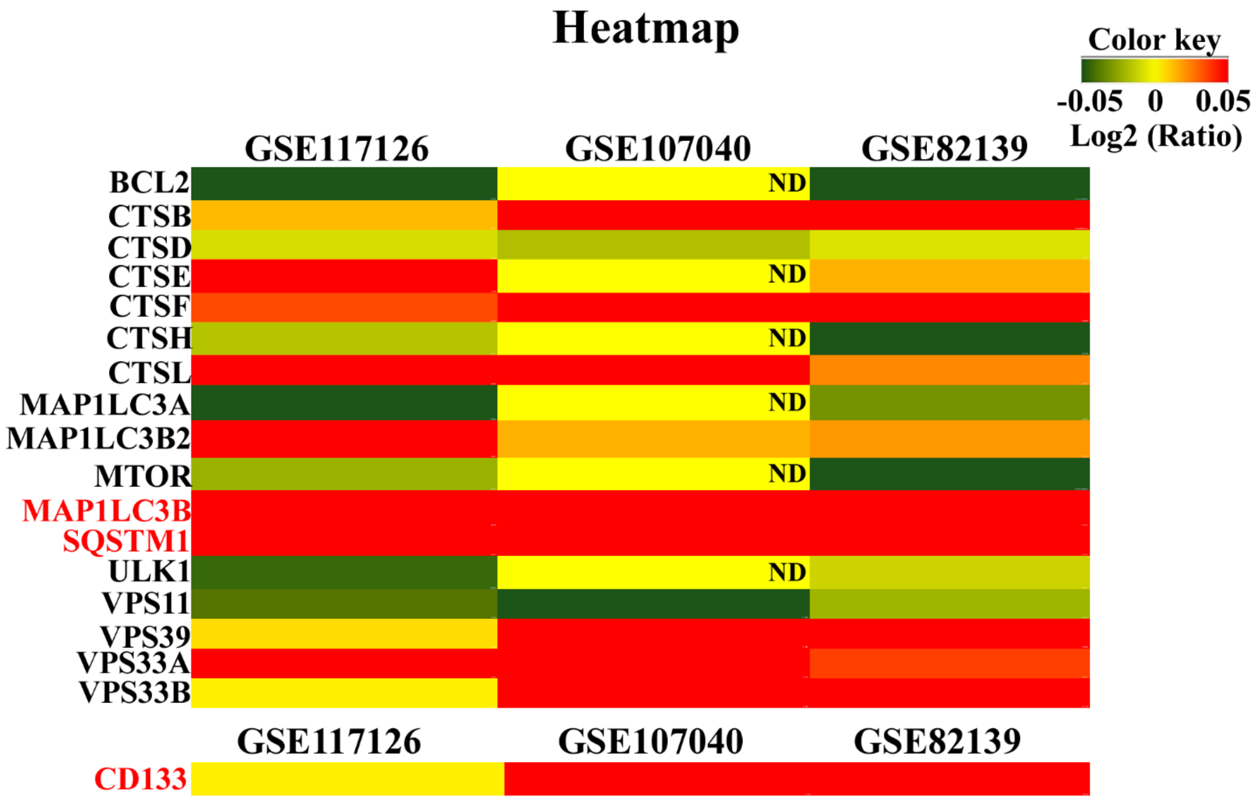

Figure S1. Heatmap of autophagy related genes and CD133 from three GSE datasets. In heatmap, we found 3 genes (MAP1LC3B, SQSTM1, CD133) related to P62.

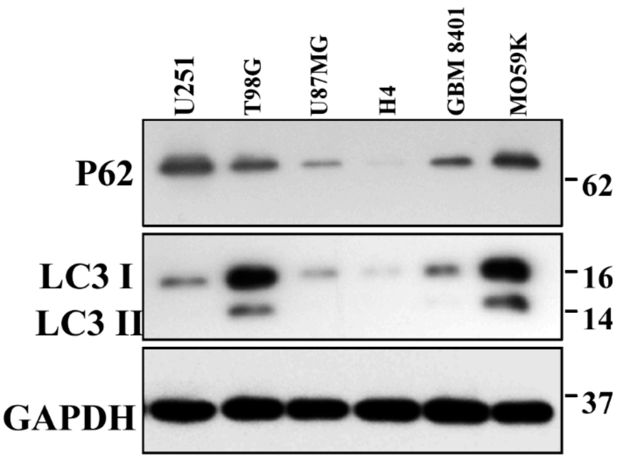

Figure S2. Western blotting analysis of P62 and LC3 I/LC3 II protein expression in glioblastoma cell lines (U251, T98G, U87MG, H4, GBM8401 and MO59K).

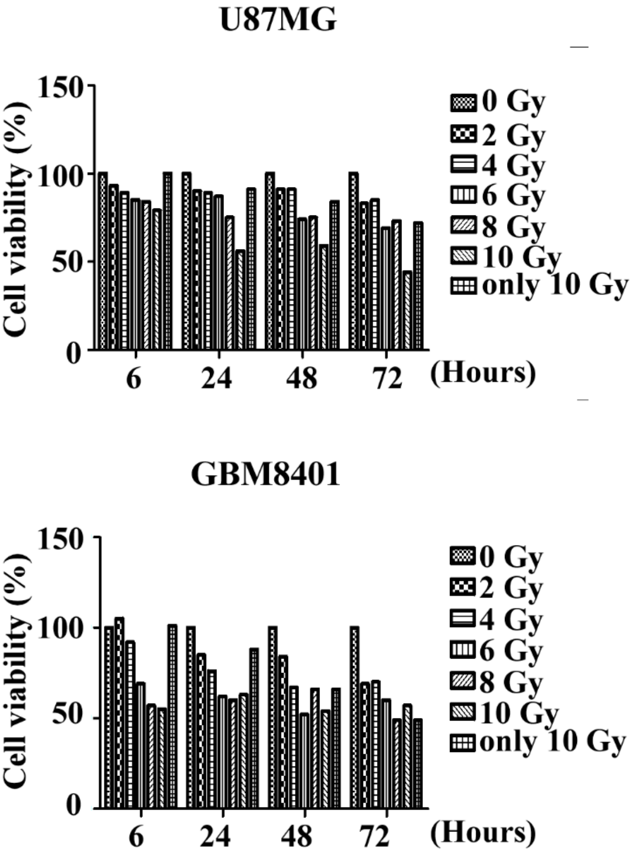

Figure S3. Cell viability in U87MG and GBM8401 after the irradiation: We designed our radiation experiments in dose- and fraction-dependent manners with 2 Gy (2 Gy per day for 1 day; total dose was 2 Gy), 4 Gy (2 Gy per day for 2 days; total dose was 4 Gy), 6 Gy (2 Gy per day for 3 days; total dose was 6 Gy), 8 Gy (2 Gy per day for 4 days; total dose was 8 Gy), 10 Gy (2 Gy per day for 5 days; total dose was 10 Gy), and 10 Gy alone for 1 day. Cells were incubated for 6, 24, 48, and 72 h in the presence of irradiated, after which cell viability was assessed using MTT assays.
